# The sodium new houttuyfonate suppresses NSCLC via activating pyroptosis through TCONS‐14036/miR‐1228‐5p/PRKCDBP pathway

**DOI:** 10.1111/cpr.13402

**Published:** 2023-01-25

**Authors:** Rilei Jiang, Bing Lu, Fanchao Feng, Qian Li, Xiaolei Chen, Shibing Cao, Zhaoxia Pan, Zhengming Deng, Yufei Zhou, Ping Liu, Jiatuo Xu

**Affiliations:** ^1^ School of Basic Medicine Science Shanghai University of Traditional Chinese Medicine Shanghai China; ^2^ Shuguang Hospital Affiliated to Shanghai University of Traditional Chinese Medicine Shanghai China; ^3^ Pulmonary and Critical Care Medicine Jiangsu Province Hospital of Traditional Chinese Medicine Nanjing Jiangsu China; ^4^ Medical Department, Shanghai First Maternity and Infant Hospital Tongji University School of Medicine Shanghai China; ^5^ Ma'an Shan Institute of Rehabilitation, Shanghai University of Traditional Chinese Medicine Shanghai China; ^6^ Department of General Surgery Jiangsu Province Hospital of Traditional Chinese Medicine Nanjing Jiangsu China; ^7^ Department of Outpatient Jiangpu Community Health Service Center Kunshan Jiangsu China; ^8^ E‐Institute of Shanghai Municipal Education Committee Shanghai University of Traditional Chinese Medicine Shanghai China

## Abstract

Several studies have suggested the potential value of *Houttuynia cordata* as a therapeutic agent in lung cancer, but direct evidence is still lacking. The study aimed to determine the regulatory impact of a major *H. cordata* constituent derivative (sodium new houttuyfonate [SNH]) on lncRNA networks in non‐small cell lung cancer (NSCLC) to identify new potential therapeutic targets. After exposing NSCLC cells to SNH, we analysed the following: cell death (via flow cytometry, TUNEL and ASC speck formation assays), immune factors (via ELISA), gene transcription (via RT‐qPCR), subcellular localisation (via FISH), gene–gene and gene‐protein interactions (via dual‐luciferase reporter and RNA immunoprecipitation assays, respectively) and protein expression and distribution (via western blotting and immunocytochemistry or immunohistochemistry). In addition, statistical analysis (via one‐way ANOVA or unpaired *t*‐tests) was performed. Exposure to SNH promoted NSCLC cell pyroptosis, concomitant with significant up‐regulation of TCONS‐14036, a novel lncRNA. Mechanistic research demonstrated that TCONS‐14036 functions as a competing endogenous (ce)RNA by sequestering microRNA (miR)‐1228‐5p, thereby up‐regulating PRKCDBP‐encoding transcript levels. Indeed, PRKCDBP promoted pyroptosis by activating the NLRP3 inflammasome, resulting in CASP1, IL‐1β and GSDMD cleavage. Our findings elucidate the potential molecular mechanisms underlying the ability of SNH to suppress NSCLC growth through activation of pyroptosis via the TCONS‐14036/miR‐1228‐5p/PRKCDBP pathway. Thus, we identify a new potential therapeutic targets for NSCLC.

## Graphical abstract

SNH to suppress NSCLC via activating pyroptosis through TCONS‐14036/miR‐1228‐5p/PRKCDBP pathway, which result in CASP1, IL‐1β and GSDMD cleavage.

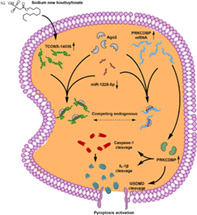

## 1 INTRODUCTION

Lung cancer is the one of the leading causes of cancer‐related deaths in worldwide.^1^ The latest statistics declared that despite the fact that lung cancer‐related deaths have decreased rapidly in the last 26 years, the number of deaths caused by lung cancer outnumber those by breast cancer, prostatic cancer and colorectal cancer combined.^2^

Pyroptosis is recognized as a highly inflammatory form of programmed cell death,^3^, ^4^ characterized by cell swelling and large bubbles emerging from the plasma membrane.^5^, ^6^ In pyroptosis, mature Caspase‐1 proteolytically cleaves the members of gasdermin family, such as GSDMD, and activates inflammatory cytokines, such as IL‐18 and IL‐1β.^7^ Pyroptosis plays a critical role in modulating the growth of cancer in vitro and in vivo.^8^

Natural products (NPs) have always been used in traditional medicine to treat diseases.^9^ According to one study of anti‐cancer drugs in modern medicine, NPs were the source of 75% approved small molecules from 1981 to 2014.^10^ Traditional Chinese medicine is a a library of NPs that has been confirmed clinically and passed down for thousands of years. The discovery of anti‐cancer small molecules in traditional Chinese medicine may be both justified and efficient.

*Houttuynia cordata* Thunb. is a traditional Chinese medicine that has been used to treat lung disease in China. The potential value of *Houttuynia cordata* in lung cancer treatment was reported in several studies.^11^, ^12^, ^13^ Research by Chen et al. declared that the *Houttuynia cordata* Thunb extract modulates G0/G1 phase arrest and Fas/CD95‐dependent death receptor apoptotic cell death in human lung cancer A549 cells, but failed to mention the critical compound.^12^ Han et al. characterized the polysaccharide structure of *Houttuynia cordata* and found that the Caspase‐3 cleavage and cyclinB1 expression was upregulated in A549 cell line.^11^ Lou et al. believed that the bioactive compound 2‐undecanone, found in *Houttuynia cordata*, could suppress the lung tumorigenesis through activating the Nrf2‐HO‐1/NQO‐1 signalling pathway.^13^

Sodium new houttuyfonate (SNH) is a ramification of Sodium houttuyfonate (SH) derived from *Houttuynia cordata*. Recent papers reported that the regulation of SH was diverse, always referring to NF‐κB signal pathways and displaying an relationship with inflammation.^14^, ^15^, ^16^ Latest papers of SNH have solely focused on its antibacterial properties and identified the mechanism of regulating Quorum Sensing^17^ or Ras1‐cAMP‐Efg1 Pathway.^18^ In our previous study, we first suggested that SNH could suppresses metastasis in non‐small cell lung cancer (NSCLC) by regulating EMT progression.^19^ However, the mechanism by which SNH direct induces NSCLC cell death is still unclear.

Recently, an increasing number of lncRNAs were reported to modulate oncological downstream DNA, RNA and protein through chromatin remodelling, transcription and post‐transcriptional regulation.^20^, ^21^, ^22^ There are still some similarities between the multiple network regulation mechanism of lncRNA and the complex regulation mechanism of traditional Chinese medicine. As a result, we attempted to find the link between lncRNA and SNH.

Herein, we first demonstrated that SNH induced the cleavage of Caspase‐1 and GSDMD, consequently inducing pyroptosis of NSCLC cells. The transcriptome sequencing screened out a novel lncRNA TCONS‐14036, which was found to regulate pyroptosis. Further analysis revealed that the TCONS‐14036 and the mRNA of PRKCDBP function as competing endogenous RNA (ceRNA) to miR‐1228‐5p. Importantly, high PRKCDBP expression is associated with the transcriptional signature of activated pyroptosis in NSCLC, implying the involvement of PRKCDBP in the anti‐oncogenic activity of pyroptosis. As a whole, our findings uncover a transcriptional and post‐transcriptional network that sustains SNH regulation of NSCLC pyroptosis via TCONS‐14036/miR‐1228‐5p/PRKCDBP pathway, suggesting a potential therapeutic target for lung cancer treatment.

## 2 MATERIALS AND METHODS

### 2.1 Reagents and cell lines

Sodium new houttuyfonate (SNH; MW: 330.41, purity ≥ 98%) was obtained from Shanghai Yuanye Bio‐technology Co. Ltd. (Shanghai, China). Dissolving SNH at 75°C ddH_2_O to get 16 mmol/L stock solution and storing at 4°C. Dose concentrations in vivo and in vitro were explored in previous studies.

HBE, NCI‐H1299, A549, SK‐MES‐1 and 293T cell lines were purchased from Stem Cell Bank, Chinese Academy of Sciences (Shanghai, China). NCI‐H23 and NCI‐H2170 were provided by Center for Traditional Chinese Medicine and Immunology Research, SHUTCM (Shanghai, China). 293T, A549, SK‐MES‐1 and HBE cells were cultured in DMEM/F12 1:1 (HyClone, USA) with 10% FBS (Gibco, Australia) and 1% P/S (Gibco, Australia). NCI‐H1299, NCI‐H23 and NCI‐H2170 were cultured in RPMI medium (HyClone) supplemented with 10% FBS (Gibco, Australia) and 1% P/S (Gibco, Australia). All the cell lines were maintained at 37°C with 5% CO_2_.

### 2.2 Plasmid construction and cell transfection

The TCONS‐14036 overexpression plasmid (p‐TCONS‐14036) and negative control (NC) plasmid (p‐NC) were constructed by pGMLV‐6395 plasmid (Table S1). Three individual short hairpin RNA plasmids for TCONS‐14036 (sh‐TCONS‐14036‐1, sh‐TCONS‐14036‐2 and sh‐TCONS‐14036‐3) and a negative control (sh‐NC) were designed by 2494‐pGMLV‐SC5 plasmid (Table S1). Plasmids including the binding sites for hsa‐miR‐1228‐5p on TCONS‐14036 and PRKCDBP mRNA were designed by pmirGLO plasmids (Table S2). The hsa‐miR‐1228‐5p mimics/inhibitors, NC mimics/inhibitors and all of the plasmids were purchased from Genomeditech Co. (Shanghai, China) (Table S3). Cell transfection was performed by the manufacturer's protocol of Lipofectamine 2000 transfection reagent (Invitrogen, USA).

### 2.3 RT‐qPCR

Total RNA was isolated with TRIzol reagent (Invitrogen). LncRNA and mRNA was performed using RevertAid First Strand cDNA Synthesis Kit (Thermo Fisher, USA) and EvaGreen 2X qRT‐PCR MasterMix‐Low ROX (abm, Canada). miRNA quantification was performed using miRNA First Strand cDNA Synthesis (Stem‐loop Method) (Sangon Biotech, Shanghai, China) and MicroRNA qPCR Kit (SYBR Green Method) (Sangon Biotech, Shanghai, China). Quantification of lncRNA, mRNA and miRNA were performed as described previously.^19^ Primer list is supplied in Table S4.

### 2.4 Western blot analysis

Cells were lysed in RIPA buffer (Solarbio, USA) with 1% PMSF (Beyotime, Shanghai, China) and analysed for total protein concentration. Fifteen milligram of total protein were suspended in SDS‐PAGE sample loading buffer (Beyotime, Shanghai, China), separated on 10%–15% SDS‐PA gels (Beyotime, Shanghai, China) and transferred onto pure PVDF membranes (Invitrogen). Primary antibodies were used as follow: Caspase‐1 (1:1000, #3866, CST, USA), Cleaved Caspase‐1 (1:1000, #4199, CST), GSDMD (1:1000, #93709, CST), Cleaved GSDMD (1:1000, #36425, CST), IL‐1β (1:1000, #12703, CST), Cleaved IL‐1β (1:1000, #83186, CST), PRKCDBP (l:200, #16250‐1‐AP, Proteintech) and β‐actin (1:2000, #4970, CST).

### 2.5 TUNEL assay analysis

A one step TUNEL Assay Kit (Beyotime, Shanghai, China) was used to perform the cell death assay. After the treatment, the cells were fixed with a 4% formaldehyde solution and permeabilized cells with Triton‐100. Staining cells with TUNEL detection buffer (4% TdT enzyme in Fluorescence‐labelled solution) for 60 min in 37°C protected from light. Mounting cells with Antifade mounting medium with DAPI (Beyotime, Shanghai, China). The cells were then observed under a Laser Scanning Confocal Microscope (Leica, TCS SP5, Germany) with excitation wavelengths of 550 nm (Cy3) and 360 nm (DAPI).

### 2.6 Flow cytometric analysis

For flow cytometric cell death assay, cells were treated with different concentrations of SNH for 24 h before collection. The cell death assays were conducted by using an Annexin V apoptosis Detection Kit with PI (BioLegend). The cell death ratio was then detected using a FACS ARIA II SORP Flow Cytometer (BD, USA).

### 2.7 ELISA

The IL‐1β and IL‐18 in cell cultured supernatant solution and animals' serums were detected by Human IL‐1β High Sensitivity ELISA Kit (EK101BHS‐01, Multi Science, Hangzhou, China) and Human IL‐18 ELISA Kit (EK118‐01, Multi Science, Hangzhou, China) according to the manufacturer's protocols. The optical density was measured at a wavelength of 450 nm, while measurement at 570 nm were screened as an internal control.

### 2.8 Immunocytochemistry

Cells were seeded on laser confocal petri dishes and exposure to indicated treatments. 5% goat serum and 0.3% Triton X‐100 in phosphate‐buffered saline (PBS) were used to block empty spaces. Incubating cells with a primary antibody against ASC/TMS1 (1:200, 10500‐1‐AP, Proteintech, USA) or NLRP3 (1:200, #ab4207, abcam, USA) in antibody dilution buffer (ADB; 1X PBS/1% bovine serum albumin (BSA)/0.3% Triton X‐100) overnight at 4°C. The next day, incubating cells with the anti‐rabbit IgG (H + L), F(ab')2 fragment (Alexa Fluor® 488 Conjugate, 1:1000, #4412, CST) or Cy3‐conjugated Affinipure Donkey Anti‐Goat IgG(H + L) (1:100, SA00009‐3, Proteintech, USA) in ADB for 2 h at room temperature protected from light. Then, Cells were incubated with Antifade Mounting Medium with DAPI (Beyotime, Shanghai, China) to detect ASC specks/NLRP3 expression and distribution using a laser scanning confocal microscope (Leica, TCS SP5, Germany).

### 2.9 RNA florescent in situ hybridization (FISH)

The RNA florescent in situ hybridization of TCONS‐14036 was performed on glass coverslips using the RNA FISH (double‐marked) Detection Reagent kit (Servicebio, Wuhan, China). The specific TCONS‐14036 probes were made by Servicebio.

### 2.10 RNA immunoprecipitation (RIP) assay

The EZ‐Magna RNA immunoprecipitation kit (Millipore, USA) was used for RIP assay. The lysates of NSCLC cells samples were incubated with magnetic beads conjugated with Ago2 antibody (#ab32381, abcam, UK) or control IgG (Millipore) overnight at 4°C. After washing three times, the beads were incubated with 0.1% SDS/0.5 mg/mL proteinase K for 30 min at 55°C. The immunocomplexes were then analysed by RT‐qPCR.

### 2.11 Dual‐luciferase reporter assays

The target genes (including TCONS‐14036/PRKCDBP wild types or mutations) were inserted into the pmirGLO Dual‐Luciferase miRNA Target Expression Vector (Promega, USA). The cells were co‐transfected with 20 pmol of miR‐mimics and 0.5 μg of pmirGLO plasmids mixed in 2 μl of Lipofectamine 2000 reagent (Invitrogen). After 24 h transfection, lysing cells to detect the firefly and renilla luciferase with Dual‐Luciferase Reporter Assay System (Promega) on the Spectra‐Max i3x microplate reader (Molecular Devices, USA). The activation of renilla luciferase recognized as an internal control.

### 2.12 Animal experiments

We constructed a NSCLC orthotopic xenograft tumour model to simulate the disease progression. First, we used lentivirus (Ubi‐MCS‐firefly_Luciferase‐IRES‐Puromycin, GENE, Shanghai, China) to establish the luciferase‐expressing NCI‐H1299 cell line. Then, we diluted the NCI‐H1299‐luc cells with Martigel 354248 (Corning, USA) and RPMI 1640 (5 × 10^6^ in 0.2 ml medium of a 1:1 mixture of RPMI 1640 and Matrigel 354248). The mixture was injected into left lung parenchyma of BALA/c nude mice (SPF, female, 1 month, 20 g) with isoflurane anaesthesia. After 2 weeks, we made a comprehensive evaluation of the NSCLC model establishment and randomly divided mice into three groups (*n* = 10). The first group regarded as model group. The mice in second group were treated with 37.5 mg/kg SNH orally (p.o.) daily. The left lung parenchyma of mice in third group were injected with 1 × 10^6^ TCONS‐14036 overexpression lentivirus (PGMLV‐CMV‐TCONS‐00014036‐EF1‐ZsGreen1‐T2A‐Puro, Genomeditech, Shanghai, China). During 21 consecutive days feeding, we monitored luciferase imaging of live animals using an IVIS Spectrum bioluminescence imaging system (PerkinElmer, USA). At the end of the feeding, we collected the blood serum to test IL‐1β and IL‐18 with ELISA kit. And we collected lung tissues to do RT‐qPCR, western blot, routine haematoxylin‐eosin (H&E) staining and immunohistochemistry staining. The protocols for the animal experiments were approved by the Ethics Review Committee of Shanghai University of Traditional Chinese Medicine (PZSHUTCM200724014).

### 2.13 Immunohistochemistry

Tissue sections were dewaxed, dehydrated and rehydrated. Then, Antigen Retrieval Citra Plus Solution (BioGenex, #HK080‐9K) was used for antigen retrieval, and hydrogen peroxide (3.0%) was used to block endogenous peroxidase activity. After blocking with 10% goat plasma, primary antibodies, including an antibody against PRKCDBP (l:200, #16250‐1‐AP, Proteintech, USA), were added to the sections and incubated at 4 °C overnight. SignalStain Antibody Diluent (CST) was used to detect the primary antibodies. Counterstaining was performed using haematoxylin.

### 2.14 Statistical analysis

Dates for the experiments were presented as the mean ± SD of three independent experiments. Differences between groups were analysed by one‐way ANOVA or unpaired *t*‐tests. *p* < 0.05 was considered as statistically significant (*p < 0.05, **p < 0.01, ***p < 0.005 and ****p < 0.001).

## 3 RESULTS

### 3.1 SNH promotes NSCLC cell pyroptosis

**FIGURE 1:**
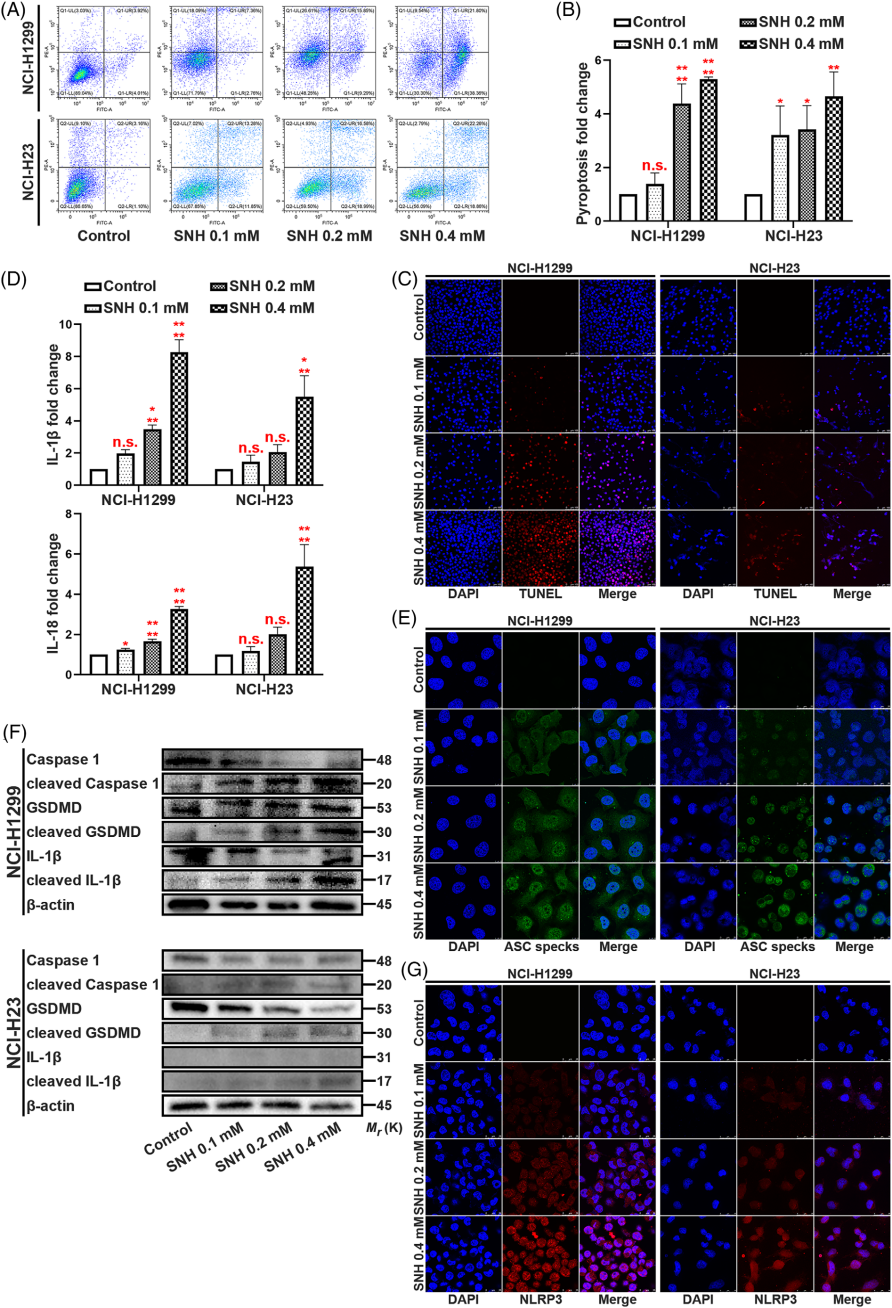
SNH promotes NSCLC cell pyroptosis. (A,B) Annexin V and PI staining flow cytometric analysis of NCI‐H1299 and NCI‐H23 cells death with 0, 0.1, 0.2 and 0.4 mM SNH treatment. (C) TUNEL stain assay in NCI‐H1299 and NCI‐H23 with the treatment of SNH. (D) ELISA assay tested the IL‐1β and IL‐18, which released in NCI‐H1299 and NCI‐H23 cells culture mediums by the treatment of SNH. (E) Immunofluorescence staining of ASC specks expression in NCI‐H1299 with the treatment of SNH. (F) Expression of pyroptosis associated proteins in NCI‐H1299 and NCI‐H23 cells with the treatment of SNH as determined by western blot analysis. (G) Immunofluorescence staining of NLRP3 expression in the treatment of SNH in NCI‐H1299 and NCI‐H23 cells. The bars and error bars indicate the mean ± SD. n.s.*p* > 0.05, *p < 0.05, **p < 0.01, ***p < 0.005 and ****p < 0.001.

In our previous study, we demonstrated that SNH suppresses NSCLC cells in vitro. The IC50 of SNH on NSCLC cell lines was 87.45–94.27 μmol/l.^19^ Herein, we tested SNH on NSCLC cell lines at the previously used dose. To understand the condition of NCI‐H1299 and NCI‐H23, we conducted several phenotype experiments. Using flow cytometric analysis, we found that the death of NSCLC cells was increased dose‐ dependently with the increase in SNH concentration (Figure 1A,B). Consistently, the TUNEL staining also revealed that the death rate of NSCLC cells was increased as the SNH dose increased (Figure 1C). Next, we used ELISA to test IL‐1β and IL‐18, the two typical pyroptosis associated inflammatory factor. Interestingly, we found that SNH increased the expression of IL‐1β and IL‐18 (Figure 1D). Further experimentation revealed that a high dosage of SNH promoted ASC oligomerization and the formation of ASC speaks (Figure 1E). Mechanism research demonstrated that SNH promoted the cleavage of Caspase‐1, GSDMD and IL‐1β (Figure 1F). Immunofluorescence staining further revealed that SNH upregulated the expression of NLRP3 and was involved in its distribution in NSCLC cells (Figure 1G). Altogether, these findings suggest that the SNH induces NSCLC cell death through pyroptosis.

### 3.2 TCONS‐14036 is a potential target of SNH

**FIGURE 2:**
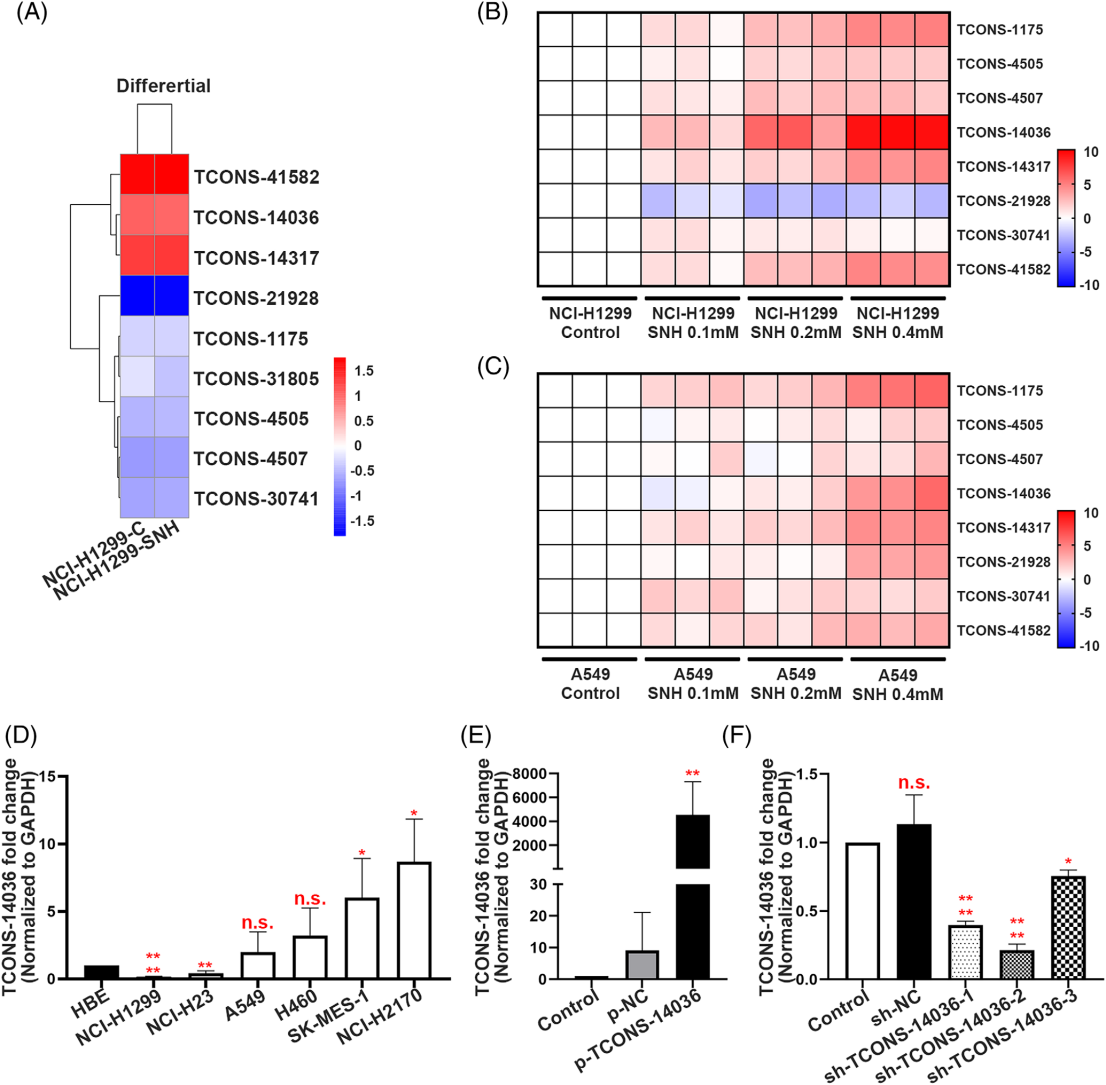
TCONS‐14036 is a potential target of SNH. (A) Heat map of the differential expression of screened novel lncRNA sequences in NCI‐H1299 cells treated or not treated with SNH for 24 H. (B,C) qRT‐PCR retrospective experiment in NCI‐H1299 and A549 cells screened out TCONS‐14036 was the most significant regulated novel lncRNA. (D) TCONS‐14036 expression in 6 NSCLC cell lines and 1 human bronchial epithelial cell line (HBE) as determined by qRT‐PCR. (E) Validation of TCONS‐14036 overexpression by p‐TCONS‐14036 in NCI‐H1299 cells as determined by qRT‐PCR. (F) Validation of sh‐RNA knockdown efficiency in NCI‐H2170 cells as determined by qRT‐PCR. The bars and error bars indicate the mean ± SD. n.s.*p* > 0.05, *p < 0.05, **p < 0.01, ***p < 0.005 and ****p < 0.001.

To identify RNAs whose biological functions are dysregulated in NSCLC with SNH, NCI‐H1299 samples were used to analyse the profile of lncRNA and mRNA transcriptome sequencing performed using SNH. In this analysis, nine unreported lncRNAs were screened out that were significantly regulated by SNH (Figure 2A). In our previous research, KEGG analysis was performed for SNH influenced mRNAs, and the inflammation associated pathways such as TNF signalling pathway, TGF‐β signalling pathway and NF‐κB signalling pathway were found to be promoted.^19^ After performing a qRT‐PCR verification on A549 and NCI‐H1299, the TCONS‐14036 was found to be the most significantly regulated unreported lncRNA (Figure 2B,C). Thus, we suspected that TCONS‐14036 was the main target regulated by SNH and the regulatory pathway may be associate with immunity. TCONS‐14036 was found to be expressed at significantly lower levels in NCI‐H1299 and NCI‐H23 than in HBE cells in a diverse range of human NSCLC cells and bronchial epithelial cells (HBE), while it was found to be highly expressed in NCI‐H2170 and SK‐MES‐1 cells (Figure 2D). To further investigate the role of TCONS‐14036 in NSCLC cells, we constructed a TCONS‐14036 overexpression vector using PGMLV‐6395 (Figure 2E). We also used pGMLV‐SC5 to construct three individual kinds of TCONS‐14036‐knockdown plasmids, named sh‐TCONS‐14036‐1, sh‐TCONS‐14036‐2 and sh‐TCONS‐14036‐3. In vitro testing revealed that the knockdown efficiency of sh‐TCONS‐14036‐2 was achieving to 70% (Figure 2F). Therefore, we used sh‐TCONS‐14036‐2 to conduct the following experiments.

### 3.3 TCONS‐14036 activates pyroptosis in NSCLC

**FIGURE 3:**
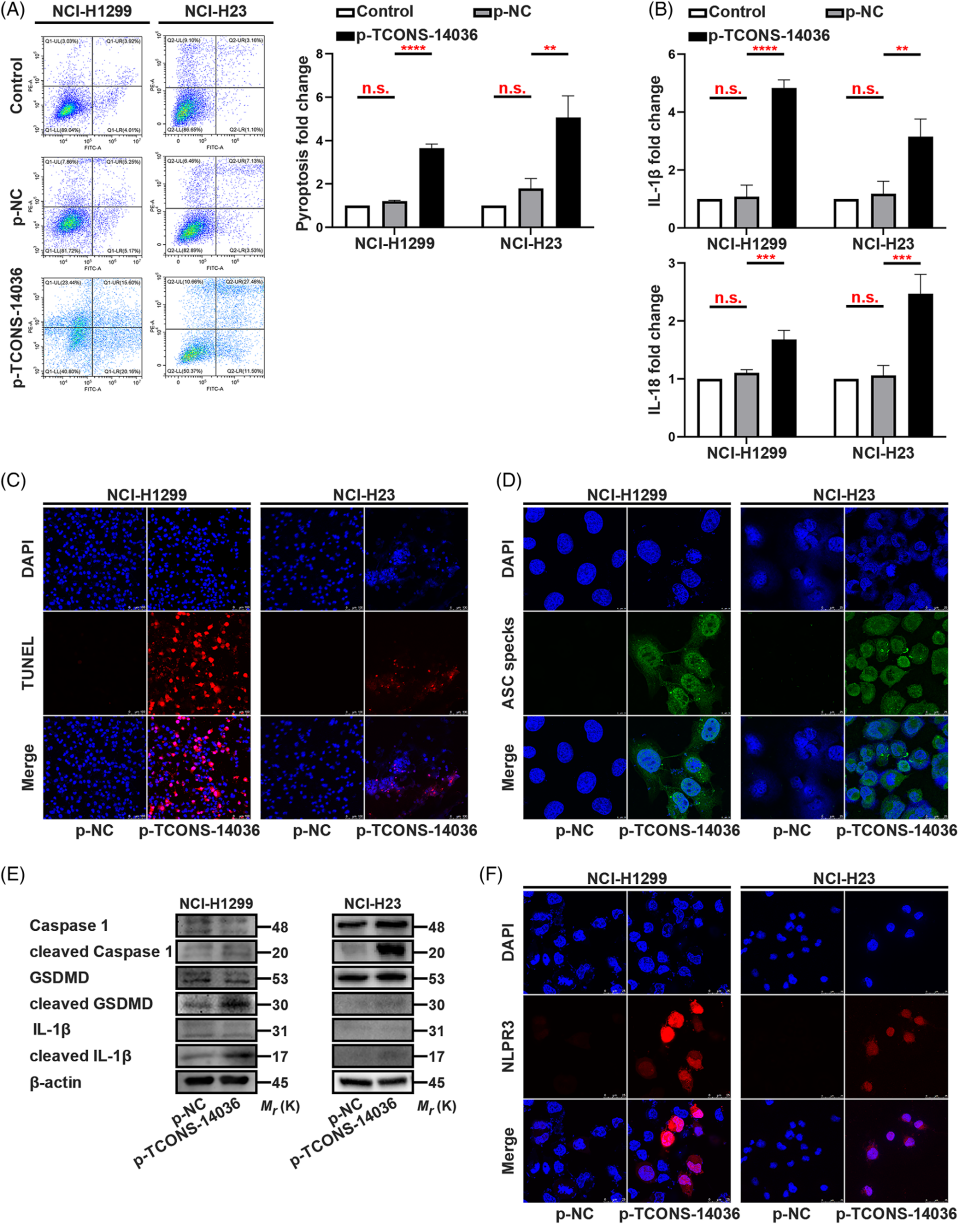
TCONS‐14036 activates pyroptosis in NSCLC. (A) Annexin V and PI staining flow cytometric analysis of NCI‐H1299 and NCI‐H23 cells death with p‐TCONS‐14036 transfection. (B) ELISA assay tested the IL‐1β and IL‐18, which released in NCI‐H1299 and NCI‐H23 cells culture mediums by p‐TCONS‐14036 transfection. (C) TUNEL stain assay in NCI‐H1299 and NCI‐H23 with the transfection of p‐TCONS‐14036. (D) Immunofluorescence staining of ASC specks expression in NCI‐H1299 and NCI‐H23 with the transfection of p‐TCONS‐14036. (E) Expression of pyroptosis associated proteins in NCI‐H1299 and NCI‐H23 cells with the transfection of p‐TCONS‐14036 as determined by western blot analysis. (F) Immunofluorescence staining of NLRP3 expression in the transfection of p‐TCONS‐14036 in NCI‐H1299 and NCI‐H23 cells. The bars and error bars indicate the mean ± SD. n.s.*p* > 0.05, *p < 0.05, **p < 0.01, ***p < 0.005 and ****p < 0.001.

To identify the relationship between TCONS‐14036 and NSCLC cells, we used Annexin V/PI flow cytometric analysis. The cell mass migrated to the right quadrant with p‐TCONS‐14036 transfection, which means that cell death was induced (Figure 3A). Using ELISA, we found that TCONS‐14036 overexpression upregulated IL‐1β and IL‐18 expression (Figure 3B). TUNEL staining revealed that p‐TCONS‐14036 induced morphological cell death (Figure 3C). The appearance of ASC specks with TCONS‐14036 overexpression was a character of pyroptosis on morphology (Figure 3D). To further confirm the biological function of TCONS‐14036, we used western blot and immunofluorescence. Western blot revealed that the cleavage of Caspase‐1, GSDMD and IL‐1β was upregulated by p‐TCONS‐14036 transfection (Figure 3E). With the successful transfection of the plasmids, immunofluorescence revealed that p‐TCONS‐14036 induced NLRP3 inflammasome high expression and diffusion (Figure 3F). Transfection of sh‐TCONS‐14036‐2 into NCI‐H2170 cells yielded a negative result (Figure S1). Treating the cells with 0.2 mM SNH and transfecting sh‐TCONS‐14036‐2 simultaneously reversed pyroptosis (Figure S2A,B). Combined, these results demonstrated that the TCONS‐14036 was required for inflammation‐mediated pyroptosis in NSCLC cell death.

### 3.4 TCONS‐14036 functions as a ceRNA and sponges miR‐1228‐5p in NSCLC cells

**FIGURE 4:**
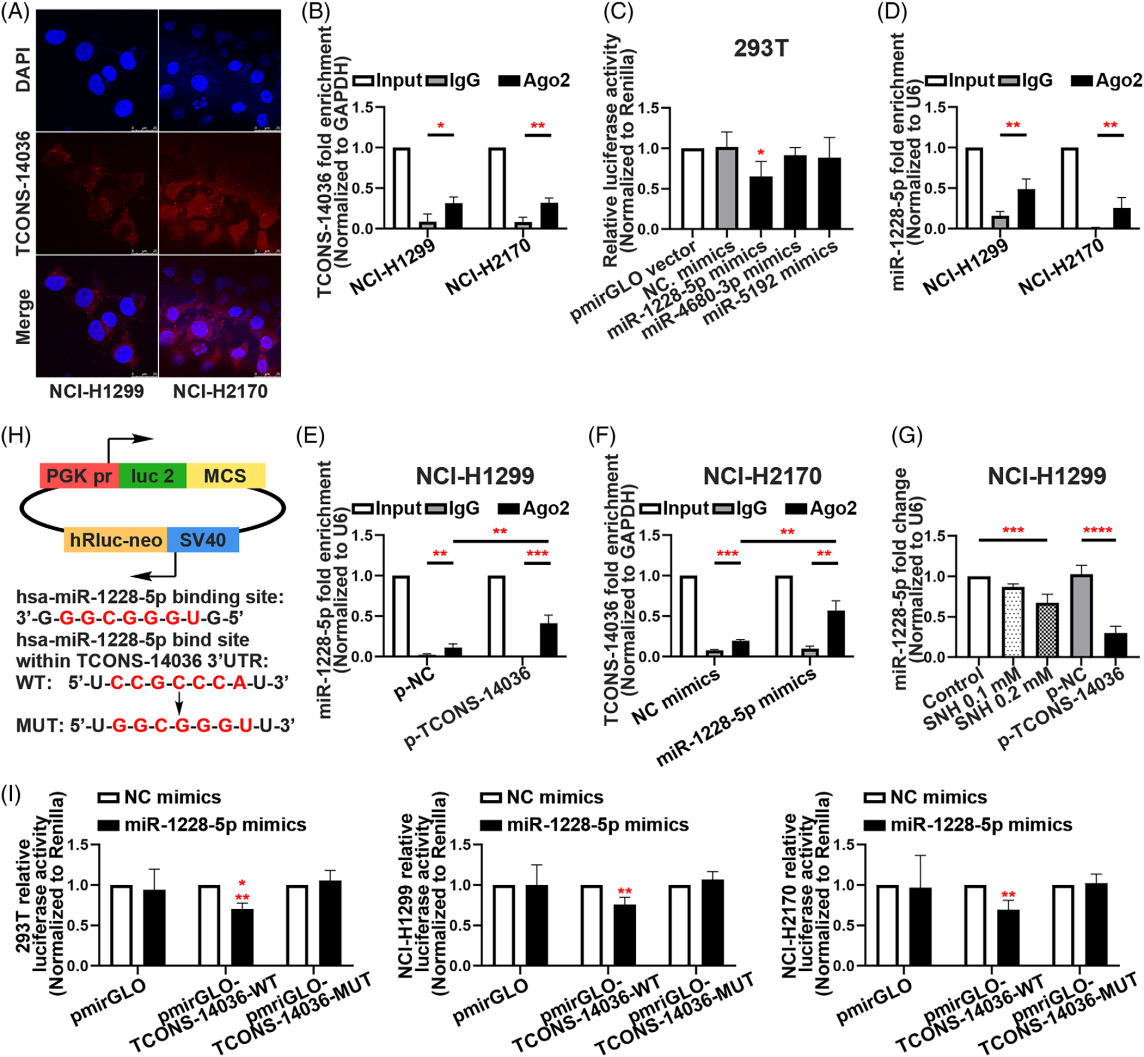
TCONS‐14036 functions as a ceRNA and sponges miR‐1228‐5p in NSCLC cells. (A) The RNA florescent in situ hybridization of TCONS‐14036 in NCI‐H1299 and NCI‐H2170 cell lines. (B) RNA immunoprecipitation (RIP) assay of TCONS‐14036 binding to Ago2 in NCI‐H1299 and NCI‐H2170 cell extracts. (C) Dual‐luciferase reporter assays were used to determine the interaction between miRNAs and TCONS‐14036. (D) RIP assay of miR‐1228‐5p binding to Ago2 in NCI‐H1299 and NCI‐H2170 cell extracts. (E) RIP assay of miR‐1228‐5p binding to Ago2 in p‐NC/p‐TCONS‐14036 transfected NCI‐H1299 cell extracts. (F) RIP assay of TCONS‐14036 binding to Ago2 in NC/miR‐1228‐5p mimics transfected NCI‐H2170 cell extracts. (G) qRT‐PCR analysis of miR‐1228‐5p in NCI‐H1299 cells after the treatment of SNH or p‐NC/p‐TCONS‐14036 transfection. (H) Predicted binding sites for miR‐1228‐5p on TCONS‐14036 and a diagram depicting the construction of the wild type (WT) and mutant type (MUT) pmirGLO‐TCONS‐14036 plasmids. (I) 293T, NCI‐H1299 and NCI‐H2170 cells were co‐transfected with miR‐1228‐5p mimics or NC mimics and pmirGLO or pmirGLO‐TCONS‐14036‐WT or pmirGLO‐TCONS‐14036‐MUT. Luciferase activity was detected 24 H after transfection using a dual‐luciferase assay. The bars and error bars indicate the mean ± SD. n.s.*p* > 0.05, *p < 0.05, **p < 0.01, ***p < 0.005 and ****p < 0.001.

To confirm the location of TCONS‐14036 in NSCLC cells, we conducted RNA florescent in situ hybridization (FISH) experiment. The results revealed the TCONS‐14036 was located in the cytoplasm (Figure 4A). Interestingly, the miRNAs were found in the cytoplasm and functionally working in the form of miRNA ribonucleoprotein complexes (miRNPs).^23^ MiRNPs was a product based on competing endogenous RNA (ceRNA) theory, which includes Ago2 sponging miRNAs and lncRNA/mRNAs.^24^, ^25^ The RNA immunoprecipitation (RIP) assay between TCON‐14036 and Ago2 protein confirmed that they could sponge together (Figure 4B). Using bioinformatic prediction tool ‘miRDB’, we found that miR‐1228‐5p, miR‐4680‐3p and miR‐5192 could combine with TCONS‐14036. Using Dual‐luciferase reporter assays identification, we found that miR‐1228‐5p had the strongest binding ability comparing to other miRNAs (Figure 4C). The RIP assay between miR‐1228‐5p and Ago2 protein verified the existence of miRNP of miR‐1228‐5p (Figure 4D). After transfecting p‐TCONS‐14036, the enrichment of miR‐1228‐5p was significant upregulated in NCI‐H1299 (Figure 4E). Consistently, the transfection of miR‐1228‐5p also led to high enrichment of TCONS‐14036 in NCI‐H2170 (Figure 4F). SNH influence or TCONS‐14036 overexpression downregulated miR‐1228‐5p expression in the cytoplasm, which verified the regulatory pathway of SNH/TCONS‐14036/miR‐1228‐5p (Figure 4G). To identify the miR‐1228‐5p binding sites on TCONS‐14036, we performed dual‐luciferase reporter assays for further transcriptional analysis. After inserting bioinformatic predicted binding site in pmirGLO plasmids, we co‐transferred the NC/wildtype/mutation plasmids and the miR‐1228‐5p/NC mimics in 293T/NCI‐H1299/NCI‐H2170 cell lines. The result exhibited that the miR‐1228‐5p mimics significantly downregulated luciferase of TCONS‐14036‐WT plasmids in all cells (Figure 4H,I). Taken together, these results validate that miR‐1228‐5p is a direct target of TCONS‐14036 in NSCLC cells.

### 3.5 Knockdown of miR‐1228‐5p induces pyroptosis in NSCLC

**FIGURE 5:**
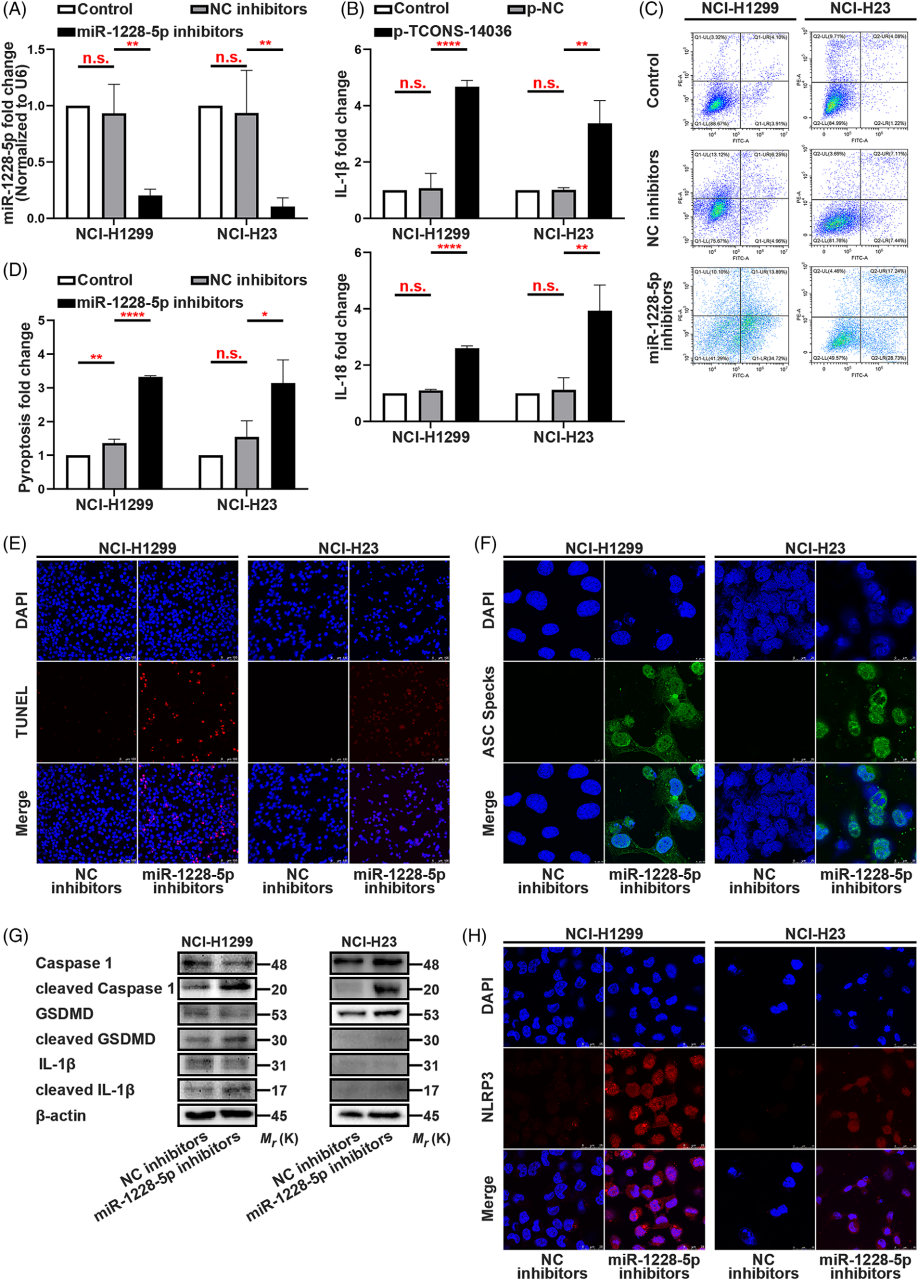
Knockdown of miR‐1228‐5p induces pyroptosis in NSCLC. (A) MiR‐1228‐5p expression in miR‐1228‐5p inhibitors transfected NCI‐H1299 and NCI‐H23 cells as determined by qRT‐PCR. (B) ELISA assay tested the IL‐1β and IL‐18, which released in NCI‐H1299 and NCI‐H23 cells culture mediums by miR‐1228‐5p inhibitors transfection. (C,D) Annexin V and PI staining flow cytometric analysis of NCI‐H1299 and NCI‐H23 cell death with miR‐1228‐5p inhibitors transfection. (E) TUNEL stain assay in NCI‐H1299 and NCI‐H23 with the transfection of miR‐1228‐5p inhibitors. (F) Immunofluorescence staining of ASC specks expression in NCI‐H1299 and NCI‐H23 with the transfection of miR‐1228‐5p inhibitors. (G) Expression of pyroptosis associated proteins in NCI‐H1299 cells with the transfection of miR‐1228‐5p inhibitors as determined by western blot analysis. (H) Immunofluorescence staining of NLRP3 expression in the transfection of miR‐1228‐5p inhibitors in NCI‐H1299 and NCI‐H23 cells. The bars and error bars indicate the mean ± SD. n.s.*p* > 0.05, *p < 0.05, **p < 0.01, ***p < 0.005 and ****p < 0.001.

To determine the biological function of miR‐1228‐5p in NSCLC pyroptosis, we identified the miR‐1228‐5p inhibitors (Figure 5A). In ELISA, the transfection of miR‐1228‐5p inhibitors increased IL‐1β and IL‐18 levels, resulting in an activated immunity (Figure 5B). Annexin V/PI flow cytometric analysis and TUNEL staining verified that downregulation of miR‐1228‐5p promoted NCI‐H1299 and NCI‐H23 cells death (Figure 5C–E). The formation of ASC specks is a feature of the pyroptosis induced by miR‐1228‐5p inhibitors (Figure 5F). Western blotting revealed that miR‐1228‐5p could positively modulate the cleavage of pyroptosis associated proteins such as, Caspase‐1, IL‐1β and GSDMD in NCI‐H1299 and NCI‐H23 cells (Figure 5G). Immunofluorescence experiment demonstrated that miR‐1228‐5p inhibition expended NLRP3 distribution in NSCLC cells (Figure 5H). Rescue experiments showed that miR‐1228‐5p‐downregulation is the critical factor of TCONS‐14036‐induced‐pyroptosis (Figure S2C,D). Therefore, we concluded that knockdown of miR‐1228‐5p induces pyroptosis in NSCLC cells.

### 3.6 miR‐1228‐5p directly targets PRKCDBP

**FIGURE 6:**
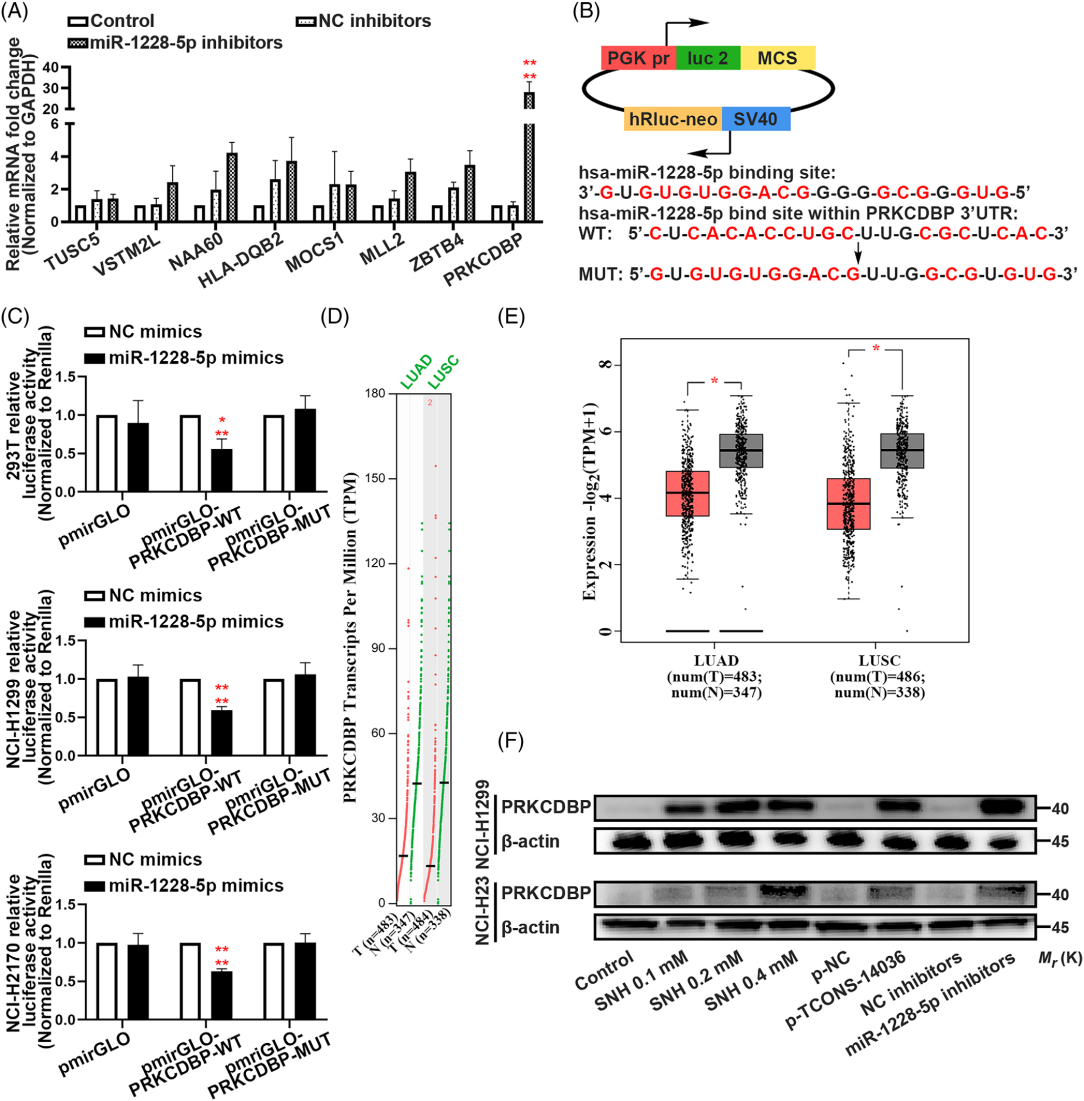
MiR‐1228‐5p directly targets PRKCDBP. (A) Verification of miR‐1228‐5p downstream targets by qRT‐PCR in NCI‐H1299 and NCI‐H23. (B) Predicted binding sites for miR‐1228‐5p on PRKCDBP and a diagram depicting the construction of the wild type (WT) and mutant type (MUT) pmirGLO‐PRKCDBP plasmids. (C) 293T, NCI‐H1299 and NCI‐H2170 cells were co‐transfected with miR‐1228‐5p mimics or NC mimics and pmirGLO or pmirGLO‐PRKCDBP‐WT or pmirGLO‐PRKCDBP‐MUT. Luciferase activity was detected 24 H after transfection using a dual‐luciferase assay. (D,E) TCGA analysis of PRKCDBP Transcripts Per Million (TPM) of lung adenocarcinoma (LUAD) and lung squamous carcinoma (LUSC). (F) Expression of PRKCDBP in NCI‐H1299 and NCI‐H23 cells with the treatment of SNH and transfection of p‐TCONS‐14036 and miR‐1228‐5p inhibitors as determined by western blot analysis. The bars and error bars indicate the mean ± SD. n.s.*p* > 0.05, *p < 0.05, **p < 0.01, ***p < 0.005 and ****p < 0.001.

To further elucidate the molecular mechanism underlying how miR‐1228‐5p exerted its effect on pyroptosis in NSCLC cells, we used bioinformatics tools (psRNATarget, December 24, 2019) to identify downstream targets. Following inclusion criteria with an expectation score of ≤5 (maximum cut‐off of score based on given scoring schema), we collected 158 potential targets (Table S5). QRT‐PCR, with more stringent inclusion criteria with an expectation score of ≤2.5, was performed to verify the potential target (including eight genes, Figure 6A). Following the variation tendency of complementary base pairing, the expression of PRKCDBP mRNA was the most significantly changed (Figure 6A). With rigorous binding site prediction between miR‐1228‐5p and PRKCDBP mRNA using bioinformatics, we constructed plasmids in the pmirGLO vector adding wild‐type (WT) or mutated‐type (MUT) binding regions of the firefly luciferase gene (Figure 6B). In 293T, NCI‐H1299 and NCI‐H2170 cell lines, co‐transfection with miR‐1228‐5p mimics and wild‐type plasmids significantly reduced luciferase activity (Figure 6C). The Cancer Genome Atlas (TCGA) analysis of lung adenocarcinoma (LUAD) and lung squamous carcinoma (LUSC) clinic samples demonstrated that the PRKCDBP transcripts per million (TPM) was low expressed in tumour tissues than in normal tissues (Figure 6D,E). Next, western blotting showed the PRKCDBP was expression was upregulated by SNH, overexpression of TCONS‐14036, and inhibition of miR‐1228‐5p (Figure 6F). Combining these results, we conclude that the miR‐1228‐5p directly targets PRKCDBP in NSCLC cells.

### 3.7 PRKCDBP overexpression confers pyroptosis via NLRP3 releasing

**FIGURE 7:**
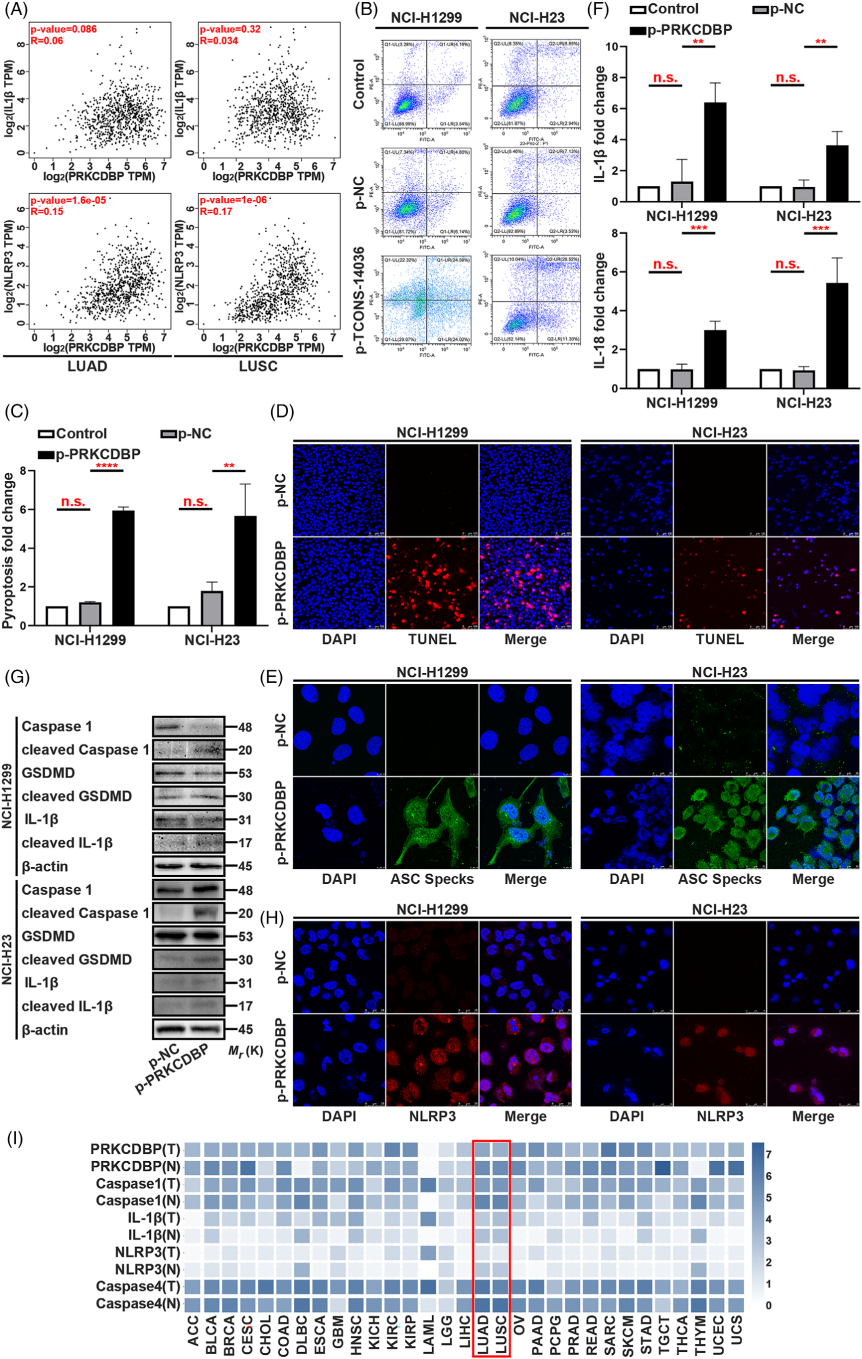
PRKCDBP overexpression confers pyroptosis. (A) Scatter plot of Transcripts Per Million (TPM) of clinic between PRKCDBP and IL‐1β or NLRP3. (B,C) Annexin V and PI staining flow cytometric analysis of NCI‐H1299 and NCI‐H23 cells death with p‐PRKCDBP transfection. (D) TUNEL stain assay in NCI‐H1299 and NCI‐H23 with the transfection of p‐PRKCDBP. (E) Immunofluorescence staining of ASC specks expression in NCI‐H1299 and NCI‐H23 with the transfection of p‐PRKCDBP. (F) ELISA assay tested the IL‐1β and IL‐18, which released in NCI‐H1299 and NCI‐H23 cells culture mediums by p‐PRKCDBP transfection. (G) Expression of pyroptosis associated proteins in NCI‐H1299 and NCI‐H23 cells with the transfection of p‐PRKCDBP as determined by western blot analysis. (H) Immunofluorescence staining of NLRP3 expression in the transfection of p‐PRKCDBP in NCI‐H1299 and NCI‐H23 cells. (I) TCGA analysis of PRKCDBP, Caspase‐1, IL‐1β, NLRP3 and Caspase‐4 expression in clinic. The bars and error bars indicate the mean ± SD. n.s.*p* > 0.05, *p < 0.05, **p < 0.01, ***p < 0.005 and ****p < 0.001.

To investigate the mechanism of PRKCDBP in NSCLC, we tried to understand the significance of IL‐1β and NLRP3 in clinical tissues from LUAD and LUSC. The R value expressed NLRP3 much more strongly (Figure 7A). Later, we conducted a phenotypic experiment using Annexin V/PI flow cytometric analysis, which exhibited the overexpression of PRKCDBP aggravated cell death (Figure 7B,C). Consistently, TUNEL and ASC immunofluorescence clearly demonstrated that the cell death was caused by pyroptosis, which was induced by PRKCDBP overexpression (Figure 7D,E). ELISA assay showed that the IL‐1β and IL‐18 expression was upregulated by PRKCDBP overexpression (Figure 7F). Furthermore, we conducted western blotting, which showed the significantly high cleavage of Caspase‐1, IL‐1β and GSDMD by p‐PRKCDBP (Figure 7G). It was interesting that the stain of NLRP3 expression had a high overlap ratio to p‐PRKCDBP transfection which may means indicate that NLRP3 and PRKCDBP are in close contact (Figure 7H). A clinic analysis of TCGA revealed that PRKCDBP, Caspase‐1, IL‐1β, NLRP3 and Caspase‐4 were expressed at low level in tumour tissues than in the normal tissues in LUAD and LUSC (Figure 7I). Taken together, these results highlighted an important role for PRKCDBP as a promotor of pyroptosis and as a regulator of the release of NLRP3.

### 3.8 SNH controls the transcriptional pathway TCONS‐14036/miR‐1228‐5p/PRKCDBP in vivo

**FIGURE 8:**
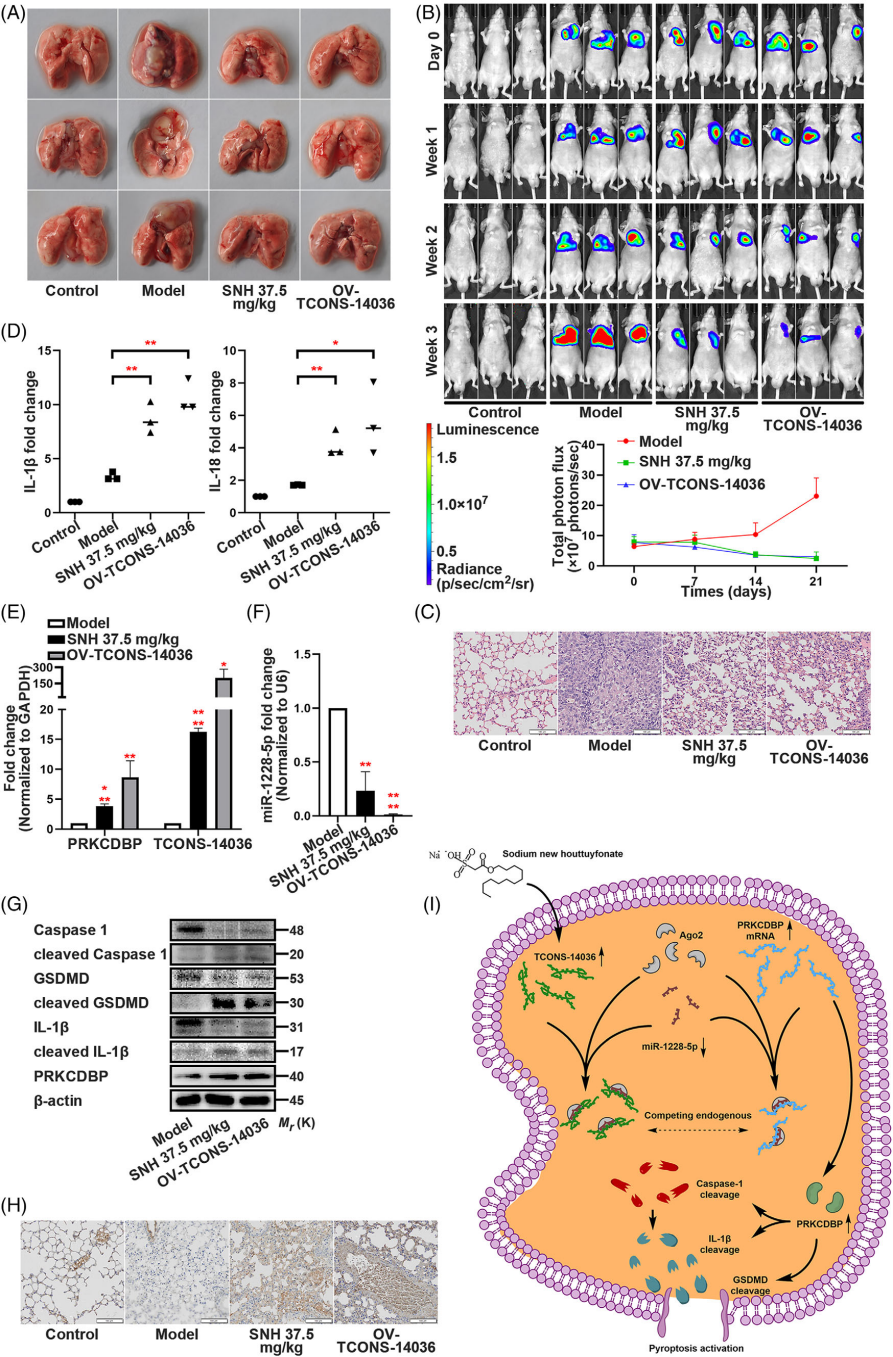
SNH controls the transcriptional pathway TCONS‐14036/miR‐1228‐5p/PRKCDBP in vivo. (A) Representative images of the lungs of nude mice which were injected with NCI‐H1299‐luc cells and treated with SNH or OV‐TCONS‐14036 lentivirus. (B) Bioluminescent imaging and quantification of photon flux of SNH or OV‐TCONS‐14036 lentivirus treatment NSCLC mice model. (C) Representative images showing haematoxylin and eosin staining of lung samples from the different groups. (D) ELISA assay tested the IL‐1β and IL‐18 which released in irrigating solutions of lungs. (E,F) PRKCDBP mRNA, TCONS‐14036 and miR‐1228‐5p levels of mice lung tissues as determined by qRT‐PCR. (G) Expression of pyroptosis associated proteins and PRKCDBP of mice lung tissues as determined by western blot analysis. (H) IHC staining showing PRKCDBP expression in the different groups. (I) Schematic diagram of mechanism on this research. The bars and error bars indicate the mean ± SD. n.s.*p* > 0.05, *p < 0.05, **p < 0.01, ***p < 0.005 and ****p < 0.001.

To analyse the SNH controlled TCONS‐14036/miR‐1228‐5p/PRKCDBP regulation pathway in vivo, we established orthotopic xenograft lung tumour model by NCI‐H1299‐luc cells. After constructing the model, the mice were fed with 37.5 mg/kg SNH (i.e., dosage supplied by previous study^19^) or orthotopically injected by TCONS‐14036 overexpression lentivirus. When compared with those of the model group, the stereological observations of the intervened groups were significantly cured (Figure 8A). Using bioluminescent images, we observed that the growth of tumours was suppressed by SNH and TCONS‐14036 and quantification of the photon flux provided the result (Figure 8B). H&E staining of the pathological sections displayed that the lung tissue was cancerous in the model group, but the symptoms were relieved with SNH or TCONS‐14036 (Figure 8C). The irrigating solutions of the lungs were tested using ELISA. When compared with those of model groups, IL‐1β and IL‐18 of SNH and TCONS‐14036 groups were activated (Figure 8D). QRT‐PCR revealed that TCONS‐14036 and PRKCDBP mRNA were upregulated by SNH and TCONS‐14036 (Figure 8E), and that the miR‐1228‐5p expression was diminished by their intervening (Figure 8F). Consistently, the western blot revealed that the cleavage of Caspase‐1, IL‐1β and GSDMD was promoted by SNH and TCONS‐14036 overexpression (Figure 8G). IHC staining revealed that SNH and TCONS‐14036 overexpression upregulated PRKCDBP expression (Figure 8H). Overall, the transcriptional pathway TCONS‐14036/miR‐1228‐5p/PRKCDBP was activated by SNH gavage and TCONS‐14036 overexpression in vivo.

## 4 DISCUSSION

Previous research identified the anti‐microbial, anti‐inflammatory and anti‐tumour properties of *Houttuynia cordata* Thunb. It has diverse biological functions, such as regulating enzyme activity, inhibiting virus activity and adjusting mRNAs and proteins in signalling pathways.^26^, ^27^ Because of its anti‐inflammatory properties, we suspected that SNH had anti‐tumour properties via an anti‐inflammatory pathway. However, the SNH ELISA produced the opposite result. SNH upregulated the inflammatory factors. Since SNH induces NSCLC cell death, we suspected that it activates an inflammation‐mediated cell death. Coincidentally, pyroptosis conformed to all characteristic. From the standpoint of phenotypic and mechanistic research, pyroptosis was confirmed to be activated by SNH. However, inflammasome sensors cannot ignore on pyroptosis. The immunofluorescence ferreted out the NLRP3, which assembled the NLRP3‐ASC‐Caspase‐1 inflammasome complex, activated by SNH.

The results of our study provide novel insights into the critical role of SNH in inducing pyroptosis of NSCLC via the TCONS‐14036/miR‐1228‐5p/PRKCDBP pathway. Herein, we detected that SNH regulated a novel lncRNA TCONS‐14036 and expressed a close relationship with inflammation pathways (Figure 2A–D). The TCONS‐14036 was a 191‐nt newfound lncRNA located at chromosome 14: 20343070–20343261 (hg38). The function of TCONS‐14036 are not reported yet. However, with its sequence being screened, the whole length of TCONS‐14036 was included in lnc‐CCNB1IP1‐1.^28^ The CCNB1IP1 (also named HEI10) is short for cyclin B1 interacting protein 1, which is a protein coding gene and widely reported to regulate meiotic recombination.^29^, ^30^ The alterations of the CCNB1IP1 ubiquitin ligases correlated significantly with breast and lung cancer prognostic factors, especially in lung cancer.^31^ Increasing evidence demonstrated that the proliferative deficiencies and transcriptional deregulation of CCNB1IP1 caused tumour mutations and invasion.^32^, ^33^ The progress free interval (PFI) of CCNB1IP1 demonstrated better prognosis in the NSCLC high expression group (Figure S3A). However, as it is a fragment of CCNB1IP1, the function of TCONS‐14036 is must be identified. By overexpression TCONS‐14036, we could easily induce pyroptosis in cells. However, after conducting a knockdown, the transfection did not have an effect. This provided the evidence of TCONS‐14036 taking part in pyroptosis of NSCLC.

Our results further revealed that TCONS‐14036 promoted pyroptosis of NSCLC by disrupting the repressive effect of miR‐1228‐5p on PRKCDBP by sponging miR‐1228‐5p. High level miR‐1228‐5p always promoted cancer progress on proliferation and metastasis.^34^, ^35^, ^36^ Survival probability of miR‐1228‐5p in LUAD and LUSC shows that decreased expression of miR‐1228‐5p improves the prognosis of NSCLC patients (Figure S3B,C).^37^ Interestingly, some research identified certain genes found in the p53 signal pathway as the targets of miR‐1228‐5p,^38^, ^39^ which proves to be consistent with the background of NCI‐H1299 and NCI‐H23 cells line in our study (The source of NCI‐H1299 and NCI‐H23 was lung cancer patient with p53 deletion). Based on the oncogenicity of miR‐1228‐5p and the bioinformatic prediction, we hypothesized that miR‐1228‐5p could be an important link between TCONS‐14036 and pyroptosis. The ceRNA theory, which based on complementary base pairing, was substantiated on the mounting lncRNA research.^40^ The dual‐luciferase report assay, FISH and RIP, helped us validate the binding site and area between TCONS‐14036 and miR‐1228‐5p. Consistently, the inhibition of miR‐1228‐5p induced pyroptosis in NSCLC cells.

The downstream regulation target of miR‐1228‐5p was also detected by combining bioinformatic prediction and binding site certification. After whole pathway verification, we believe that PRKCDBP is the target gene that regulate pyroptosis. PRKCDBP is a putative tumour suppressor whose alteration has been observed in several human cancers.^41^ Experiments in the latest oncology research regarding PRKCDBP as a transcriptional target of TNF‐α demonstrated that low expression of PRKCDBP forebode poor prognosis in tumour patients, including those with lung adenocarcinoma.^42^, ^43^ In colorectal cancers, PRKCDBP induction by TNF‐α was confirmed to be disrupted when NF‐κB signalling was blocked, and previous research revealed PRKCDBP is implicated in TNF‐α‐induced apoptosis.^41^ However, in our opinion, the cell death induced by inflammation expressed a closer relationship with pyroptosis. It is worth noting that the frequency of hypermethylation was high for ASC and PRKCDBP in a previously conducted microarray analysis in lung cancers.^44^ ASC oligomerization is a typical feature of pyroptosis. Thus, we evaluated the relationship between PRKCDBP and pyroptosis and successfully found that upregulation PRKCDBP activates pyroptosis in NSCLC cells. Using in vivo experimental approaches, we identified the key regulation points of TCONS‐14036/miR‐1228‐5p/PRKCDBP pathway and that tumours were suppressed by SNH.

In summary, we believe that the SNH directly suppressed NSCLC through activating pyroptosis. Further research revealed a novel transcriptional and post‐transcriptional network in which pyroptosis is induced by upregulation of TCONS‐14036, downregulation of miR‐1228‐5p and activation of PRKCDBP (Figure 8I). Activated factors include NLRP3 release, IL‐1β/Caspase‐1/GSDMD cleavage and ASC oligomerization. This research not only provide a theoretical basis for SNH usage in NSCLC in clinic, but also uncovered potential target for oncology therapy.

## AUTHOR CONTRIBUTIONS

Jiatuo Xu and Ping Liu directed and supervised the study; Rilei Jiang designed the experiments and wrote the manuscript; Bing Lu and Fanchao Feng performed most of the experiment; Qian Li collected and analysed the data; Xiaolei Chen completed the figures; Shibing Cao, Zhaoxia Pan, Zhengming Deng and Yufei Zhou participated in some experiments. All authors read and approved the final manuscript.

## CONFLICT OF INTEREST

The authors declare that they have no competing interests.

## Supporting information


**Figure S1.** Expression of pyroptosis associated proteins in NCI‐H2170 cells with the transfection of sh‐TCONS‐14036‐2 as determined by western blot analysis.

**Figure S2.** Rescue experiments of TCONS‐14036 and miR‐1228‐5p on pyroptosis. (A,B) Immunofluorescence staining of ASC specks (A) and ELISA assay tested the IL‐1β and IL‐18 (B) in NCI‐H1299 and NCI‐H23 with the SNH 0.2 mM treatment and sh‐TCONS‐14036‐2 transfection at the same time. (C,D) Immunofluorescence staining of ASC specks (C) and ELISA assay tested the IL‐1β and IL‐18 (D) in NCI‐H1299 and NCI‐H23 with co‐transfection of p‐TCONS‐14036 and miR‐1228‐5p mimics. (E) Verification of PRKCDBP mRNA by qRT‐PCR in NCI‐H1299 and NCI‐H23 with co‐transfection of p‐TCONS‐14036 and miR‐1228‐5p mimics.

**Figure S3.** KM plot of CCNB1IP1 and miR‐1228‐5p in NSCLC. (A) The Progress Free Interval (PFI) of CCNB1IP1 in NSCLC. (B,C) The Overall Survival (OS) of miR‐1228‐5p in LUAD (B) and LUSC (C).

**Table S1.** The p‐RNA and sh‐RNA sequence.

**Table S2.** The sequence of mimics and inhibitors.

**Table S3.** The dual‐luciferase reporter plasmids.

**Table S4.** Primers for RT‐qPCR.

**Table S5.** Basic information and bioinformatics about TCONS‐14036.

**Table S6.** The bioinformatic prediction of targets.

## Data Availability

The data used to support the fundings of this study are available from the corresponding author upon request.
