# Supplementary figures and images for: The sodium new houttuyfonate suppresses NSCLC via activating pyroptosis through TCONS‐14036/miR‐1228‐5p/PRKCDBP pathway
Source: Cell Prolif. 2023 Jan 25;56(7):e13402. doi: 10.1111/cpr.13402 (PMC10334279; doi:10.1111/cpr.13402)

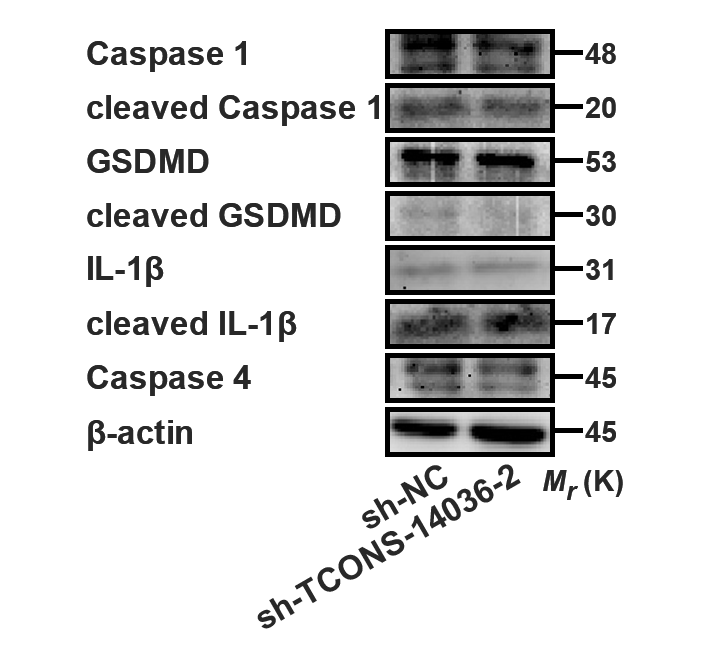

Supplement: Supplementary file 1 — Figure S1. Expression of pyroptosis associated proteins in NCI‐H2170 cells with the transfection of sh‐TCONS‐14036‐2 as determined by western blot analysis. [file CPR-56-e13402-s006.tif]

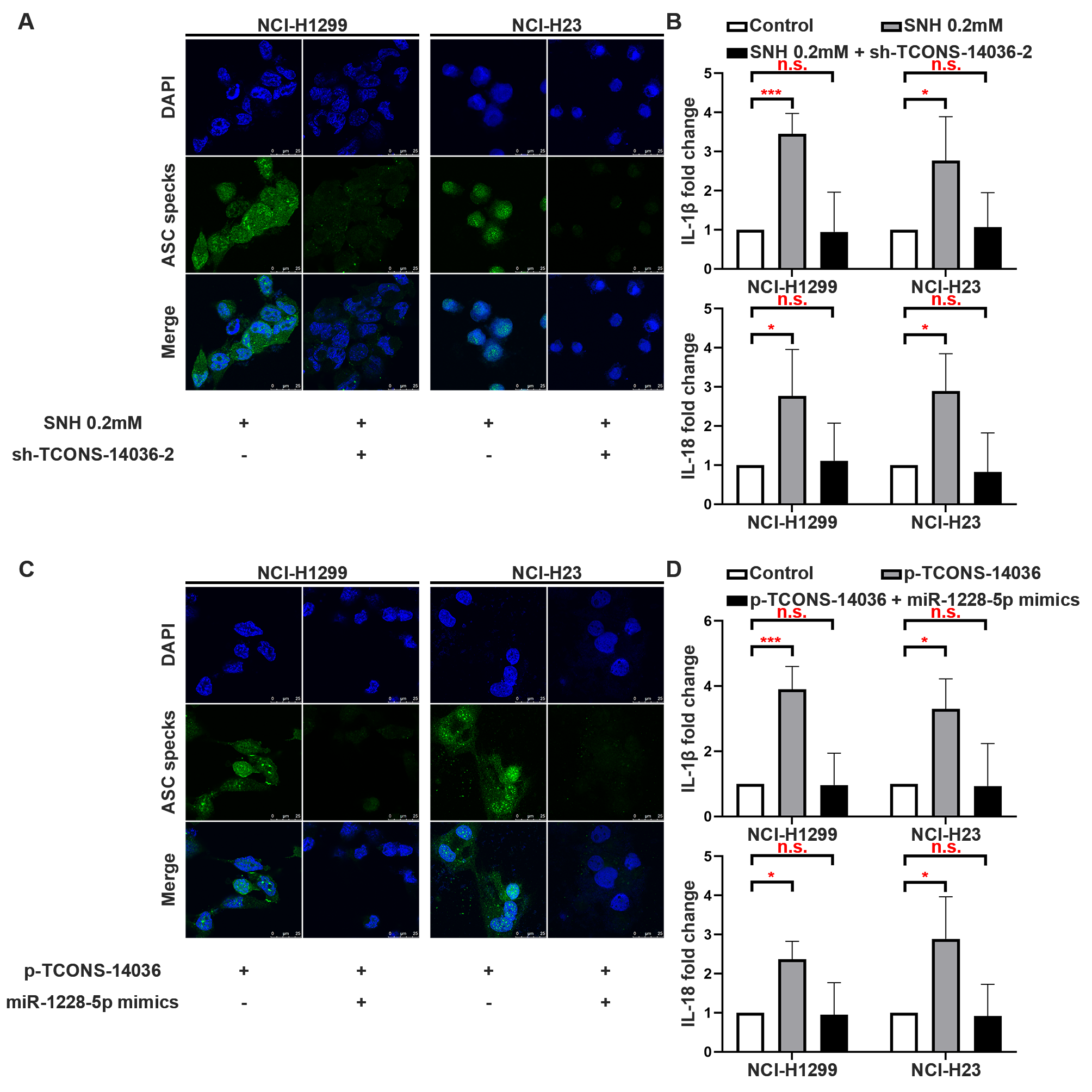

Supplement: Supplementary file 2 — Figure S2. Rescue experiments of TCONS‐14036 and miR‐1228‐5p on pyroptosis. (A,B) Immunofluorescence staining of ASC specks (A) and ELISA assay tested the IL‐1β and IL‐18 (B) in NCI‐H1299 and NCI‐H23 with the SNH 0.2 mM treatment and sh‐TCONS‐14036‐2 transfection at the same time. (C,D) Immunofluorescence staining of ASC specks (C) and ELISA assay tested the IL‐1β and IL‐18 (D) in NCI‐H1299 and NCI‐H23 with co‐transfection of p‐TCONS‐14036 and miR‐1228‐5p mimics. (E) Verification of PRKCDBP mRNA by qRT‐PCR in NCI‐H1299 and NCI‐H23 with co‐transfection of p‐TCONS‐14036 and miR‐1228‐5p mimics. [file CPR-56-e13402-s004.tif]

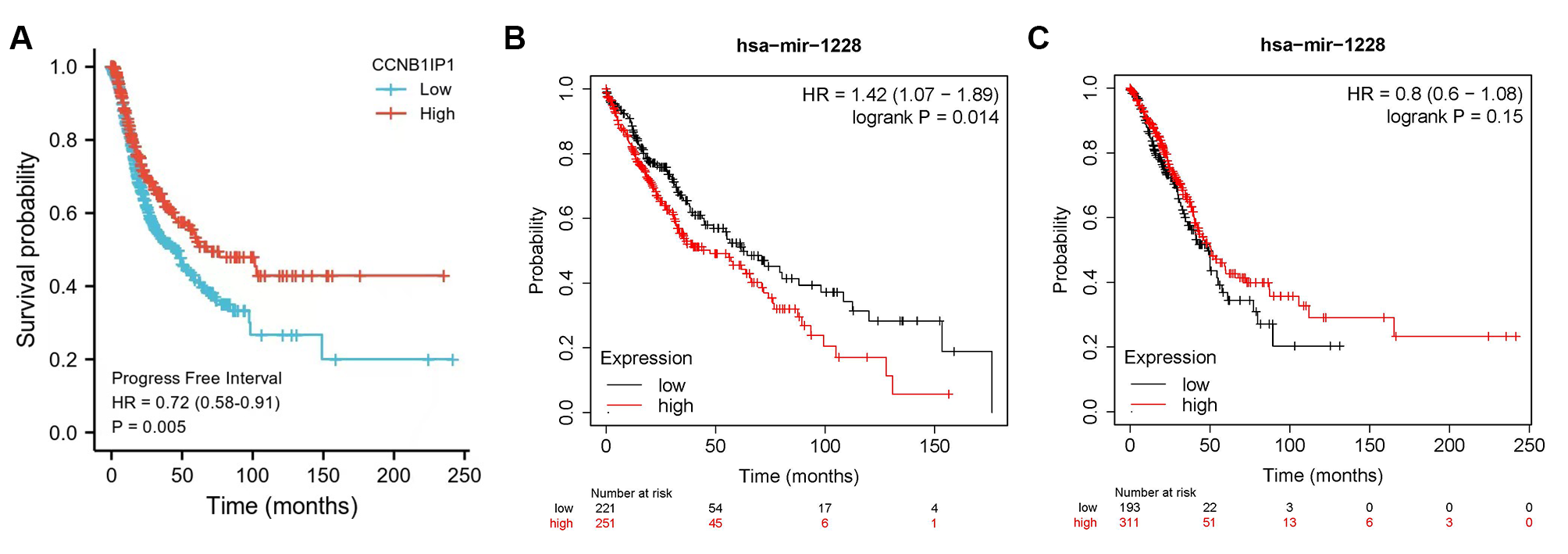

Supplement: Supplementary file 3 — Figure S3. KM plot of CCNB1IP1 and miR‐1228‐5p in NSCLC. (A) The Progress Free Interval (PFI) of CCNB1IP1 in NSCLC. (B,C) The Overall Survival (OS) of miR‐1228‐5p in LUAD (B) and LUSC (C). [file CPR-56-e13402-s002.tif]
