# Supplementary material for: The sodium new houttuyfonate suppresses NSCLC via activating pyroptosis through TCONS‐14036/miR‐1228‐5p/PRKCDBP pathway
Source: Cell Prolif. 2023 Jan 25;56(7):e13402. doi: 10.1111/cpr.13402 (PMC10334279; doi:10.1111/cpr.13402)
Supplement: Supplementary file 4 — Table S1. The p‐RNA and sh‐RNA sequence. [file CPR-56-e13402-s001.docx]

**Table S1 The p-RNA and sh-RNA sequence.**

| **Name** | **Oligo sequences** |
| --- | --- |
| P-TCONS-14036 | 5'- GGGGCTCCGCGCGAGGTCAGACTGGGCAGGAGATGCCG  TGGACCCCGCCCTTCGGGGAGGGGCCCGGCGGATGCCTCCTTTGCCGGAGCTTGGAACAGACTCACGGCCAGCGAAGTGAGTTCAATGGCTGAGGTGAGGTACCCCGCAGGGGACCTCATAACCCAATTCAGACTACTCTCCTCCGCCCATTT-3’ |
| Sh-NC-sense | 5'- gatctGTTCTCCGAACGTGTCACGTTTCAAGAGAACGTGA  CACGTTCGGAGAATTTTTTc-3’ |
| Sh-NC-antisense | 5'- aattgAAAAAATTCTCCGAACGTGTCACGTTCTCTTGAAA  CGTGACACGTTCGGAGAACa-3’ |
| Sh-TCONS-14036-1-sense | 5'- GATCCGGAGCTTGGAACAGACTCACGCTCGAGCGTGAG  TCTGTTCCAAGCTCCTTTTTT-3’ |
| Sh-TCONS-14036-1-antisense | 5'- AATTAAAAAAGGAGCTTGGAACAGACTCACGCTCGAGC  GTGAGTCTGTTCCAAGCTCCG-3’ |
| Sh-TCONS-14036-2-sense | 5'- GATCCGCCAGCGAAGTGAGTTCAATGCTCGAGCATTGA  ACTCACTTCGCTGGCTTTTTT-3’ |
| Sh-TCONS-14036-2-antisense | 5'- AATTAAAAAAGCCAGCGAAGTGAGTTCAATGCTCGAGC  ATTGAACTCACTTCGCTGGCG-3’ |
| Sh-TCONS-14036-3-sense | 5'- GATCCGGGACCTCATAACCCAATTCACTCGAGTGAATTG  GGTTATGAGGTCCCTTTTTT-3’ |
| Sh-TCONS-14036-3-antisense | 5'- AATTAAAAAAGGGACCTCATAACCCAATTCACTCGAGT  GAATTGGGTTATGAGGTCCCG-3’ |
| P-PRKCDBP | 5'- GCCACCATGAGGGAGAGTGCGTTGGAGCGGGGGCCTGT  GCCCGAGGCGCCGGCGGGGGGTCCCGTGCACGCCGTGACGGTGGTGACCCTGCTGGAGAAGCTGGCCTCCATGCTGGAGACTCTGCGGGAGCGGCAGGGAGGCCTGGCTCGAAGGCAGGGAGGCCTGGCAGGGTCCGTGCGCCGCATCCAGAGCGGCCTGGGCGCTCTGAGTCGCAGCCACGACACCACCAGCAACACCTTGGCGCAGCTGCTGGCCAAGGCGGAGCGCGTGAGCTCGCACGCCAACGCCGCCCAAGAGCGCGCGGTGCGCCGCGCAGCCCAGGTGCAGCGGCTGGAGGCCAACCACGGGCTGCTGGTGGCGCGCGGGAAGCTCCACGTTCTGCTCTTCAAGGAGGAGGGTGAAGTCCCAGCCAGCGCTTTCCAGAAGGCACCAGAGCCCTTGGGCCCGGCGGACCAGTCCGAGCTGGGCCCAGAGCAGCTGGAGGCCGAAGTTGGAGAGAGCTCGGACGAGGAGCCGGTGGAGTCCAGGGCCCAGCGGCTGCGGCGCACCGGATTGCAGAAGGTACAGAGCCTCCGAAGGGCCCTTTCGGGCCGGAAAGGCCCTGCAGCGCCACCGCCCACCCCGGTCAAGCCGCCTCGCCTTGGGCCTGGCCGGAGCGCTGAAGCCCAGCCGGAAGCCCAGCCTGCGCTGGAGCCCACGCTGGAGCCAGAGCCTCCGCAGGACACCGAGGAAGATCCCGGGAGACCTGGGGCTGCCGAAGAAGCTCTGCTCCAAATGGAGAGTGTAGCCTGA-3’ |
