# Supplementary material for: The sodium new houttuyfonate suppresses NSCLC via activating pyroptosis through TCONS‐14036/miR‐1228‐5p/PRKCDBP pathway
Source: Cell Prolif. 2023 Jan 25;56(7):e13402. doi: 10.1111/cpr.13402 (PMC10334279; doi:10.1111/cpr.13402)
Supplement: Supplementary file 5 — Table S2. The sequence of mimics and inhibitors. [file CPR-56-e13402-s008.docx]

**Table S2 The sequence of mimics and inhibitors.**

| **Name** | **Sequence（5’-3’）** | | **5’mark** | **Modification** |
| --- | --- | --- | --- | --- |
| hsa-miR-1228-5p mimics | | GUGGGCGGGGGCAGGUGUGUG | N/A | N/A |
| hsa-miR-1228-5p inhibitors | | CACACACCUGCCCCCGCCCAC | N/A | 2’-oMe |
| NC mimics | | UCACAACCUCCUAGAAAGAGUAGA | N/A | N/A |
| NC inhibitors | | UCUACUCUUUCUAGGAGGUUGUGA | N/A | 2’-oMe |
| hsa-miR-4680-3p mimics | | UCUGAAUUGUAAGAGUUGUUA | N/A | N/A |
| hsa-miR-5192 mimics | | AGGAGAGUGGAUUCCAGGUGGU | N/A | N/A |
