# Supplementary material for: The sodium new houttuyfonate suppresses NSCLC via activating pyroptosis through TCONS‐14036/miR‐1228‐5p/PRKCDBP pathway
Source: Cell Prolif. 2023 Jan 25;56(7):e13402. doi: 10.1111/cpr.13402 (PMC10334279; doi:10.1111/cpr.13402)
Supplement: Supplementary file 6 — Table S3. The dual‐luciferase reporter plasmids. [file CPR-56-e13402-s007.docx]

**Table S3 The dual-luciferase reporter plasmids.**

| **Name** | **Plasmids sequences** |
| --- | --- |
| PmirGLO-TCONS-14036-WT | GCTAGCGGGGCTCCGCGCGAGGTCAGACTGGGCAGGAGATGCCGTGGACCCCGCCCTTCGGGGAGGGGCCCGGCGGATGCCTCCTTTGCCGGAGCTTGGAACAGACTCACGGCCAGCGAAGTGAGTTCAATGGCTGAGGTGAGGTACCCCGCAGGGGACCTCATAACCCAATTCAGACTACTCTCCT**CCGCCCA**TTTCTCGAG |
| PmirGLO-TCONS-14036-MUT | GCTAGCGGGGCTCCGCGCGAGGTCAGACTGGGCAGGAGATGCCGTGGACCCCGCCCTTCGGGGAGGGGCCCGGCGGATGCCTCCTTTGCCGGAGCTTGGAACAGACTCACGGCCAGCGAAGTGAGTTCAATGGCTGAGGTGAGGTACCCCGCAGGGGACCTCATAACCCAATTCAGACTACTCTCCT**GGCGGGT**TTTCTCGAG |
| PmirGLO-PRKCDBP-WT | GCTAGCCTGGGCCCAGAGCAGCTGGAGGCCGAAGTTGGAGAGAGCTCGGACGAGGAGCCGGTGGAGTCCAGGGCCCAGCGGCTGCGGCGCACCGGATTGCAGAAGGTACAGAGCCTCCGAAGGGCCCTTTCGGGCCGGAAAGGCCCTGCAGCGCCACCGCCCACCCCGGTCAAGCCGCCTCGCCTTGGGCCTGGCCGGAGCGCTGAAGCCCAGCCGGAAGCCCAGCCTGCGCTGGAGCCCACGCTGGAGCCAGAGCCTCCGCAGGACACCGAGGAAGATCCCGGGAGACCTGGGGCTGCCGAAGAAGCTCTGCTCCAAATGGAGAGTGTAGCCTGAGGGCTGGTGTTGCCTGCCTCCCCTGTGCTTGTGCCTTGTCCCAAAATAAATCCTTTCAGAATGTAGCACTCACGCCCTAATAAGGAGCGAATCCTACATCCACCAAGGCGGGCGCTCTGGCCCTCCCTTCCTTAAGCCCAGTCCTGTGTCCTCTGAAAGAGGTGCAGTCA**C**T**CACACCTGC**TTG**CGC**T**CAC**CATCAATAAAAGTAATTTCACCCGAACTCGAG |
| PmirGLO-PRKCDBP-MUT | GCTAGCCTGGGCCCAGAGCAGCTGGAGGCCGAAGTTGGAGAGAGCTCGGACGAGGAGCCGGTGGAGTCCAGGGCCCAGCGGCTGCGGCGCACCGGATTGCAGAAGGTACAGAGCCTCCGAAGGGCCCTTTCGGGCCGGAAAGGCCCTGCAGCGCCACCGCCCACCCCGGTCAAGCCGCCTCGCCTTGGGCCTGGCCGGAGCGCTGAAGCCCAGCCGGAAGCCCAGCCTGCGCTGGAGCCCACGCTGGAGCCAGAGCCTCCGCAGGACACCGAGGAAGATCCCGGGAGACCTGGGGCTGCCGAAGAAGCTCTGCTCCAAATGGAGAGTGTAGCCTGAGGGCTGGTGTTGCCTGCCTCCCCTGTGCTTGTGCCTTGTCCCAAAATAAATCCTTTCAGAATGTAGCACTCACGCCCTAATAAGGAGCGAATCCTACATCCACCAAGGCGGGCGCTCTGGCCCTCCCTTCCTTAAGCCCAGTCCTGTGTCCTCTGAAAGAGGTGCAGTCA**G**T**GTGTGGACG**TTG**GCG**T**GTG**CATCAATAAAAGTAATTTCACCCGAACTCGAG |
