# Supplementary material for: The sodium new houttuyfonate suppresses NSCLC via activating pyroptosis through TCONS‐14036/miR‐1228‐5p/PRKCDBP pathway
Source: Cell Prolif. 2023 Jan 25;56(7):e13402. doi: 10.1111/cpr.13402 (PMC10334279; doi:10.1111/cpr.13402)
Supplement: Supplementary file 7 — Table S4. Primers for RT‐qPCR. [file CPR-56-e13402-s005.docx]

**Table S4 Primers for RT-qPCR.**

| Gene | Primers for qRT-PCR |
| --- | --- |
| TCONS-1175-F | CTTGGAGCGTGTTAGGCGAGTG |
| TCONS-1175-R | GGGGCTGGGGTCACGATCC |
| TCONS-4505-F | CTCCTATCTGATGTATCTGGCGGTAAC |
| TCONS-4505-R | GGGACAATACACAGTAAGACAACTTGC |
| TCONS-4507-F | AGCAAGCCTAACTCAAGCCATTGG |
| TCONS-4507-R | TTGAAAAGAGGGGAGAGAAGCACTAAC |
| TCONS-14036-F | CCGTGGACCCCGCCCTTC |
| TCONS-14036-R | CCTCACCTCAGCCATTGAACTCAC |
| TCONS-14317-F | AGGTCACCATATTGATGCCGAACTTAG |
| TCONS-14317-R | AGGATCGCTTGAGCCCAGGAG |
| TCONS-21928-F | GTGTGCTGGACTGTGGTTGAGAC |
| TCONS-21928-R | AAGTTCTTCCGCATTCCTACACCTTAG |
| TCONS-30741-F | GACATCTACAACCTTCGATCTCCTGAC |
| TCONS-30741-R | ACTTCCAACACCCGCATTCATCC |
| TCONS-41582-F | GCGATCTGGCTGCGACATCTG |
| TCONS-41582-R | GGAGCGGTGAGGGAGGAAGG |
| TUSC5-F | CTCCTATGCCCAAGACCAAGAAGC |
| TUSC5-R | CCGTCCACGTTGCCCTGTTG |
| VSTM2L-F | GGTGGTGGGCAGCAACATCTC |
| VSTM2L-R | GCCTTGCCGTCGCTGAAGTC |
| NAA60-F | GTCTGGGCGTCGTGAAAGAGTTC |
| NAA60-R | TTGCTGTGTTGTTGGTGGTGAGG |
| HLA-DQB2-F | CCGTGATGCTGGTGATGCTGAG |
| HLA-DQB2-R | CGCTCTGTCCCGTTGGTGAAG |
| MOCS1-F | CCATCAACATCAGCCTGGACACC |
| MOCS1-R | CCTCGCATCACCACACAGTTCAC |
| MLL2-F | TGCCAGCGGTTCCTTCCTATCC |
| MLL2-R | ACAGCGAGCCTCCTCCAGATATG |
| ZBTB4-F | GCGTCCTGGCTGCTTCAAGTC |
| ZBTB4-R | AGGAAGAGGAGGAGGAGGAAGAGG |
| PRKCDBP-F | GCTCCACGTTCTGCTCTTCAAGG |
| PRKCDBP-R | TCTCTCCAACTTCGGCCTCCAG |
| Caspase1-F | CACACCGCCCAGAGCACAAG |
| Caspase1-R | TCCCACAAATGCCTTCCCGAATAC |
| IL-1β-F | CTCACAGCAGCATCTCGACAAGAG |
| IL-1β-R | TCCACGGGCAAGACATAGGTAGC |
| NLRP3-F | AGAGCCCCGTGAGTCCCATTAAG |
| NLRP3-R | CGCCCAGTCCAACATCATCTTCC |
| Caspase4-F | GGAGGCTGGACCACCTGAGTC |
| Caspase4-R | AGGCGTGTGCGGTTGTTTCTC |
| mir-1228-5p-F | AGTGGGCGGGGGCAGG |
| mir-1228-5p-R | AGTGCAGGGTCCGAGGTATT |
| mir-1228-5p-RT | GTCGTATCCAGTGCAGGGTCCGAGGTATTCGCACTGGATACGACCACACA |
| U6-F | AGAGAAGATTAGCATGGCCCCTG |
| U6-R | ATCCAGTGCAGGGTCCGAGG |
| U6-RT | GTCGTATCCAGTGCAGGGTCCGAGGTATTCGCACTGGATACGACAAAATA |
