# Supplementary material for: The sodium new houttuyfonate suppresses NSCLC via activating pyroptosis through TCONS‐14036/miR‐1228‐5p/PRKCDBP pathway
Source: Cell Prolif. 2023 Jan 25;56(7):e13402. doi: 10.1111/cpr.13402 (PMC10334279; doi:10.1111/cpr.13402)
Supplement: Supplementary file 8 — Table S5. Basic information and bioinformatics about TCONS‐14036. [file CPR-56-e13402-s003.docx]

**Table S5 The bioinformatic prediction of targets.**

| miRNA_Acc. | Target_Acc. | Expectation | UPE$ | miRNA_start | miRNA_end | Target_start | Target_end |
| --- | --- | --- | --- | --- | --- | --- | --- |
| GUGGGCGGGGGCAGGUGUGUG | NM_172367\|TUSC5 | 2 | 15.566 | 1 | 21 | 1366 | 1386 |
| GUGGGCGGGGGCAGGUGUGUG | NM_080607\|VSTM2L | 2.5 | 10.749 | 1 | 21 | 4 | 24 |
| GUGGGCGGGGGCAGGUGUGUG | NM_024845\|NAA60 | 2.5 | 14.375 | 1 | 21 | 1619 | 1639 |
| GUGGGCGGGGGCAGGUGUGUG | NM_001083600\|NAA60 | 2.5 | 14.375 | 1 | 21 | 1597 | 1617 |
| GUGGGCGGGGGCAGGUGUGUG | NM_001198858\|HLA-DQB2 | 2.5 | 9.811 | 1 | 21 | 45 | 65 |
| GUGGGCGGGGGCAGGUGUGUG | NM_005943\|MOCS1 | 2.5 | 15.999 | 1 | 21 | 1470 | 1490 |
| GUGGGCGGGGGCAGGUGUGUG | NM_003482\|MLL2 | 2.5 | 24.787 | 1 | 21 | 450 | 470 |
| GUGGGCGGGGGCAGGUGUGUG | NM_020899\|ZBTB4 | 2.5 | 20.711 | 1 | 21 | 483 | 503 |
| GUGGGCGGGGGCAGGUGUGUG | NM_145040\|PRKCDBP | 2.5 | 15.736 | 1 | 21 | 171 | 191 |
| GUGGGCGGGGGCAGGUGUGUG | NM_022658\|HOXC8 | 3 | 16.694 | 1 | 21 | 262 | 282 |
| GUGGGCGGGGGCAGGUGUGUG | NM_145272\|C17orf50 | 3 | 11.99 | 1 | 21 | 281 | 301 |
| GUGGGCGGGGGCAGGUGUGUG | NM_001013690\|FIGNL2 | 3 | 23.157 | 1 | 21 | 886 | 906 |
| GUGGGCGGGGGCAGGUGUGUG | NM_032741\|AGPAT1 | 3 | 19.064 | 1 | 21 | 589 | 609 |
| GUGGGCGGGGGCAGGUGUGUG | NM_006411\|AGPAT1 | 3 | 19.064 | 1 | 21 | 589 | 609 |
| GUGGGCGGGGGCAGGUGUGUG | NM_014830\|ZBTB39 | 3 | 11.693 | 1 | 21 | 204 | 224 |
| GUGGGCGGGGGCAGGUGUGUG | NM_006035\|CDC42BPB | 3 | 16.735 | 1 | 21 | 911 | 931 |
| GUGGGCGGGGGCAGGUGUGUG | NM_001185100\|CD22 | 3 | 13.549 | 1 | 21 | 446 | 466 |
| GUGGGCGGGGGCAGGUGUGUG | NM_005985\|SNAI1 | 3 | 10.574 | 1 | 21 | 23 | 43 |
| GUGGGCGGGGGCAGGUGUGUG | NM_003647\|DGKE | 3 | 13.866 | 1 | 21 | 5708 | 5728 |
| GUGGGCGGGGGCAGGUGUGUG | NM_001185101\|CD22 | 3 | 13.549 | 1 | 21 | 277 | 297 |
| GUGGGCGGGGGCAGGUGUGUG | NM_153274\|BEST4 | 3 | 8.296 | 1 | 21 | 2 | 22 |
| GUGGGCGGGGGCAGGUGUGUG | NM_014656\|KIAA0040 | 3 | 18.794 | 1 | 21 | 2126 | 2146 |
| GUGGGCGGGGGCAGGUGUGUG | NM_001033560\|DYX1C1 | 3 | 7.254 | 1 | 21 | 113 | 133 |
| GUGGGCGGGGGCAGGUGUGUG | NM_014747\|RIMS3 | 3.5 | 16.352 | 1 | 21 | 1276 | 1296 |
| GUGGGCGGGGGCAGGUGUGUG | NM_080927\|DCBLD2 | 3.5 | 19.817 | 1 | 21 | 2799 | 2819 |
| GUGGGCGGGGGCAGGUGUGUG | NM_014601\|EHD2 | 3.5 | 17.585 | 1 | 21 | 56 | 76 |
| GUGGGCGGGGGCAGGUGUGUG | NM_001102559\|PPAPDC1B | 3.5 | 11.845 | 1 | 21 | 434 | 454 |
| GUGGGCGGGGGCAGGUGUGUG | NM_001481\|GAS8 | 3.5 | 19.294 | 1 | 21 | 1031 | 1051 |
| GUGGGCGGGGGCAGGUGUGUG | NM_001161520\|COG5 | 3.5 | 14.709 | 1 | 21 | 489 | 509 |
| GUGGGCGGGGGCAGGUGUGUG | NM_001164695\|IYD | 3.5 | 14.64 | 1 | 21 | 4666 | 4686 |
| GUGGGCGGGGGCAGGUGUGUG | NM_001164694\|IYD | 3.5 | 14.64 | 1 | 21 | 4579 | 4599 |
| GUGGGCGGGGGCAGGUGUGUG | NM_203395\|IYD | 3.5 | 14.64 | 1 | 21 | 4479 | 4499 |
| GUGGGCGGGGGCAGGUGUGUG | NM_001013257\|BCAM | 3.5 | 8.136 | 1 | 21 | 1509 | 1529 |
| GUGGGCGGGGGCAGGUGUGUG | NM_145173\|DIRAS1 | 3.5 | 6.411 | 1 | 21 | 9 | 30 |
| GUGGGCGGGGGCAGGUGUGUG | NM_021926\|ALX4 | 3.5 | 22.051 | 1 | 21 | 296 | 316 |
| GUGGGCGGGGGCAGGUGUGUG | NM_032482\|DOT1L | 3.5 | 19.261 | 1 | 21 | 387 | 407 |
| GUGGGCGGGGGCAGGUGUGUG | NM_001037553\|AGPAT3 | 3.5 | 21.064 | 1 | 21 | 1812 | 1832 |
| GUGGGCGGGGGCAGGUGUGUG | NM_005581\|BCAM | 3.5 | 8.136 | 1 | 21 | 411 | 431 |
| GUGGGCGGGGGCAGGUGUGUG | NM_025161\|C17orf70 | 3.5 | 24.055 | 1 | 21 | 502 | 524 |
| GUGGGCGGGGGCAGGUGUGUG | NM_001130924\|TMEM201 | 3.5 | 19.966 | 1 | 21 | 141 | 161 |
| GUGGGCGGGGGCAGGUGUGUG | NM_032271\|TRAF7 | 3.5 | 22.137 | 1 | 21 | 216 | 236 |
| GUGGGCGGGGGCAGGUGUGUG | NM_212472\|PRKAR1A | 3.5 | 15.406 | 1 | 21 | 746 | 766 |
| GUGGGCGGGGGCAGGUGUGUG | NM_001037\|SCN1B | 3.5 | 10.931 | 1 | 21 | 527 | 547 |
| GUGGGCGGGGGCAGGUGUGUG | NM_001145399\|MPPED2 | 3.5 | 9.681 | 1 | 21 | 3438 | 3458 |
| GUGGGCGGGGGCAGGUGUGUG | NM_001128826\|NCS1 | 3.5 | 20.507 | 1 | 21 | 762 | 782 |
| GUGGGCGGGGGCAGGUGUGUG | NM_033133\|CNP | 3.5 | 19.928 | 1 | 21 | 3225 | 3245 |
| GUGGGCGGGGGCAGGUGUGUG | NM_015366\|PRR5 | 3.5 | 17.41 | 1 | 21 | 363 | 383 |
| GUGGGCGGGGGCAGGUGUGUG | NM_001804\|CDX1 | 3.5 | 23.234 | 1 | 21 | 443 | 463 |
| GUGGGCGGGGGCAGGUGUGUG | NM_177402\|SYT2 | 3.5 | 24.883 | 1 | 21 | 4351 | 4371 |
| GUGGGCGGGGGCAGGUGUGUG | NM_021161\|KCNK10 | 3.5 | 13.842 | 1 | 21 | 1740 | 1760 |
| GUGGGCGGGGGCAGGUGUGUG | NM_005934\|MLLT1 | 3.5 | 17.853 | 1 | 21 | 106 | 126 |
| GUGGGCGGGGGCAGGUGUGUG | NM_207291\|USF2 | 3.5 | 20.212 | 1 | 21 | 14 | 34 |
| GUGGGCGGGGGCAGGUGUGUG | NM_001193451\|TMTC1 | 3.5 | 14.282 | 1 | 21 | 774 | 794 |
| GUGGGCGGGGGCAGGUGUGUG | NM_005883\|APC2 | 3.5 | 20.233 | 1 | 21 | 2042 | 2062 |
| GUGGGCGGGGGCAGGUGUGUG | NM_001126054\|CASK | 3.5 | 14.039 | 1 | 21 | 1806 | 1826 |
| GUGGGCGGGGGCAGGUGUGUG | NM_000757\|CSF1 | 3.5 | 13.694 | 1 | 21 | 830 | 849 |
| GUGGGCGGGGGCAGGUGUGUG | NM_001042646\|TRAK1 | 3.5 | 20.09 | 1 | 21 | 438 | 458 |
| GUGGGCGGGGGCAGGUGUGUG | NM_173642\|RIMKLA | 4 | 17.955 | 1 | 21 | 1040 | 1060 |
| GUGGGCGGGGGCAGGUGUGUG | NM_001256798\|C20orf112 | 4 | 11.926 | 1 | 21 | 1986 | 2006 |
| GUGGGCGGGGGCAGGUGUGUG | NM_001130831\|GAS7 | 4 | 15.504 | 1 | 21 | 6469 | 6490 |
| GUGGGCGGGGGCAGGUGUGUG | NM_015307\|FAM189A1 | 4 | 6.022 | 1 | 21 | 2907 | 2927 |
| GUGGGCGGGGGCAGGUGUGUG | NM_004442\|EPHB2 | 4 | 6.598 | 1 | 21 | 1 | 21 |
| GUGGGCGGGGGCAGGUGUGUG | NM_024602\|HECTD3 | 4 | 13.472 | 1 | 21 | 843 | 863 |
| GUGGGCGGGGGCAGGUGUGUG | NM_001039397\|TBC1D28 | 4 | 18.204 | 1 | 21 | 593 | 613 |
| GUGGGCGGGGGCAGGUGUGUG | NM_001013257\|BCAM | 4 | 6.752 | 1 | 21 | 1167 | 1187 |
| GUGGGCGGGGGCAGGUGUGUG | NM_144775\|SMCR8 | 4 | 14.623 | 1 | 21 | 969 | 989 |
| GUGGGCGGGGGCAGGUGUGUG | NM_015085\|RAP1GAP2 | 4 | 14.635 | 1 | 21 | 171 | 191 |
| GUGGGCGGGGGCAGGUGUGUG | NM_001077418\|TMEM231 | 4 | 18.212 | 1 | 21 | 595 | 615 |
| GUGGGCGGGGGCAGGUGUGUG | NM_178518\|TMEM102 | 4 | 12.667 | 1 | 21 | 149 | 169 |
| GUGGGCGGGGGCAGGUGUGUG | NM_004040\|RHOB | 4 | 17.61 | 1 | 21 | 1183 | 1203 |
| GUGGGCGGGGGCAGGUGUGUG | NM_020748\|INTS2 | 4 | 8.494 | 1 | 21 | 1137 | 1157 |
| GUGGGCGGGGGCAGGUGUGUG | NM_005581\|BCAM | 4 | 6.607 | 1 | 21 | 69 | 89 |
| GUGGGCGGGGGCAGGUGUGUG | NM_004285\|H6PD | 4 | 6.865 | 1 | 21 | 604 | 624 |
| GUGGGCGGGGGCAGGUGUGUG | NM_181425\|FXN | 4 | 18.885 | 1 | 21 | 4438 | 4458 |
| GUGGGCGGGGGCAGGUGUGUG | NM_000144\|FXN | 4 | 18.885 | 1 | 21 | 4388 | 4408 |
| GUGGGCGGGGGCAGGUGUGUG | NM_001142505\|ABCG4 | 4 | 17.062 | 1 | 21 | 893 | 913 |
| GUGGGCGGGGGCAGGUGUGUG | NM_014388\|DIEXF | 4 | 17.863 | 1 | 21 | 4162 | 4182 |
| GUGGGCGGGGGCAGGUGUGUG | NM_001034173\|ALDH1L2 | 4 | 20.462 | 1 | 21 | 3047 | 3067 |
| GUGGGCGGGGGCAGGUGUGUG | NM_001177998\|SLC34A2 | 4 | 22.133 | 1 | 21 | 1689 | 1708 |
| GUGGGCGGGGGCAGGUGUGUG | NM_014505\|KCNMB4 | 4 | 7.83 | 1 | 21 | 645 | 665 |
| GUGGGCGGGGGCAGGUGUGUG | NM_001270614\|DEDD2 | 4 | 19.715 | 1 | 21 | 353 | 373 |
| GUGGGCGGGGGCAGGUGUGUG | NM_001040424\|PRDM15 | 4 | 20.739 | 1 | 21 | 831 | 851 |
| GUGGGCGGGGGCAGGUGUGUG | NM_133448\|TMEM132D | 4 | 16.091 | 1 | 21 | 1889 | 1909 |
| GUGGGCGGGGGCAGGUGUGUG | NM_001271891\|RGS7BP | 4 | 17.094 | 1 | 21 | 2100 | 2120 |
| GUGGGCGGGGGCAGGUGUGUG | NM_014732\|KIAA0513 | 4 | 21.528 | 1 | 21 | 3906 | 3926 |
| GUGGGCGGGGGCAGGUGUGUG | NM_001029875\|RGS7BP | 4 | 17.094 | 1 | 21 | 2012 | 2032 |
| GUGGGCGGGGGCAGGUGUGUG | NM_032959\|POLR2J2 | 4 | 20.046 | 1 | 21 | 252 | 272 |
| GUGGGCGGGGGCAGGUGUGUG | NM_001145299\|EXOC7 | 4 | 20.977 | 1 | 21 | 470 | 490 |
| GUGGGCGGGGGCAGGUGUGUG | NM_001097615\|POLR2J3 | 4 | 20.046 | 1 | 21 | 252 | 272 |
| GUGGGCGGGGGCAGGUGUGUG | NM_001114139\|EPB49 | 4 | 10.074 | 1 | 21 | 864 | 884 |
| GUGGGCGGGGGCAGGUGUGUG | NM_002501\|NFIX | 4 | 21.775 | 1 | 21 | 3638 | 3658 |
| GUGGGCGGGGGCAGGUGUGUG | NM_173598\|KSR2 | 4.5 | 5.029 | 1 | 21 | 3358 | 3379 |
| GUGGGCGGGGGCAGGUGUGUG | NM_004442\|EPHB2 | 4.5 | 21.71 | 1 | 21 | 1141 | 1161 |
| GUGGGCGGGGGCAGGUGUGUG | NM_004442\|EPHB2 | 4.5 | 18.532 | 1 | 21 | 1587 | 1607 |
| GUGGGCGGGGGCAGGUGUGUG | NM_001013257\|BCAM | 4.5 | 17.899 | 1 | 21 | 939 | 958 |
| GUGGGCGGGGGCAGGUGUGUG | NM_006650\|CPLX2 | 4.5 | 15.884 | 1 | 21 | 2697 | 2717 |
| GUGGGCGGGGGCAGGUGUGUG | NM_003369\|UVRAG | 4.5 | 21.403 | 1 | 21 | 2135 | 2155 |
| GUGGGCGGGGGCAGGUGUGUG | NM_001079871\|HAP1 | 4.5 | 24.753 | 1 | 21 | 1964 | 1984 |
| GUGGGCGGGGGCAGGUGUGUG | NM_001079871\|HAP1 | 4.5 | 18.343 | 1 | 21 | 28 | 48 |
| GUGGGCGGGGGCAGGUGUGUG | NM_006854\|KDELR2 | 4.5 | 24.949 | 1 | 21 | 1739 | 1759 |
| GUGGGCGGGGGCAGGUGUGUG | NM_001100603\|KDELR2 | 4.5 | 24.949 | 1 | 21 | 1564 | 1584 |
| GUGGGCGGGGGCAGGUGUGUG | NM_002055\|GFAP | 4.5 | 12.074 | 1 | 21 | 1241 | 1261 |
| GUGGGCGGGGGCAGGUGUGUG | NM_175922\|PRR18 | 4.5 | 21.979 | 1 | 21 | 247 | 267 |
| GUGGGCGGGGGCAGGUGUGUG | NM_002697\|POU2F1 | 4.5 | 17.643 | 1 | 21 | 849 | 869 |
| GUGGGCGGGGGCAGGUGUGUG | NM_014388\|DIEXF | 4.5 | 9.106 | 1 | 21 | 3804 | 3824 |
| GUGGGCGGGGGCAGGUGUGUG | NM_021259\|TMEM8A | 4.5 | 22.391 | 1 | 21 | 165 | 185 |
| GUGGGCGGGGGCAGGUGUGUG | NM_001184906\|FBXL20 | 4.5 | 13.419 | 1 | 21 | 160 | 180 |
| GUGGGCGGGGGCAGGUGUGUG | NM_004952\|EFNA3 | 4.5 | 22.421 | 1 | 21 | 129 | 149 |
| GUGGGCGGGGGCAGGUGUGUG | NM_001177998\|SLC34A2 | 4.5 | 14.683 | 1 | 21 | 1308 | 1328 |
| GUGGGCGGGGGCAGGUGUGUG | NM_001024956\|SC5D | 4.5 | 14.537 | 1 | 21 | 2170 | 2190 |
| GUGGGCGGGGGCAGGUGUGUG | NM_021045\|ZNF248 | 4.5 | 17.224 | 1 | 21 | 853 | 873 |
| GUGGGCGGGGGCAGGUGUGUG | NM_020988\|GNAO1 | 4.5 | 21.235 | 1 | 21 | 324 | 344 |
| GUGGGCGGGGGCAGGUGUGUG | NM_001267606\|ZNF248 | 4.5 | 17.224 | 1 | 21 | 521 | 541 |
| GUGGGCGGGGGCAGGUGUGUG | NM_006056\|NMUR1 | 4.5 | 24.918 | 1 | 21 | 58 | 78 |
| GUGGGCGGGGGCAGGUGUGUG | NM_004711\|SYNGR1 | 4.5 | 12.761 | 1 | 21 | 54 | 74 |
| GUGGGCGGGGGCAGGUGUGUG | NM_003841\|TNFRSF10C | 4.5 | 23.166 | 1 | 21 | 120 | 140 |
| GUGGGCGGGGGCAGGUGUGUG | NM_001113239\|HIPK2 | 4.5 | 24.97 | 1 | 21 | 3605 | 3625 |
| GUGGGCGGGGGCAGGUGUGUG | NM_182964\|NAV2 | 4.5 | 23.863 | 1 | 21 | 1051 | 1071 |
| GUGGGCGGGGGCAGGUGUGUG | NM_018330\|KIAA1598 | 4.5 | 10.809 | 1 | 21 | 2218 | 2238 |
| GUGGGCGGGGGCAGGUGUGUG | NM_178310\|SNAI3 | 4.5 | 24.408 | 1 | 21 | 557 | 576 |
| GUGGGCGGGGGCAGGUGUGUG | NM_033512\|TSPYL5 | 4.5 | 21.055 | 1 | 21 | 1825 | 1845 |
| GUGGGCGGGGGCAGGUGUGUG | NM_001127211\|KIAA1598 | 4.5 | 10.809 | 1 | 21 | 2007 | 2027 |
| GUGGGCGGGGGCAGGUGUGUG | NM_003087\|SNCG | 4.5 | 21.895 | 1 | 21 | 104 | 124 |
| GUGGGCGGGGGCAGGUGUGUG | NM_001010858\|RNF187 | 4.5 | 17.034 | 1 | 21 | 1675 | 1695 |
| GUGGGCGGGGGCAGGUGUGUG | NM_024735\|FBXO31 | 5 | 19.988 | 1 | 21 | 709 | 729 |
| GUGGGCGGGGGCAGGUGUGUG | NM_018043\|ANO1 | 5 | 13.959 | 1 | 21 | 698 | 719 |
| GUGGGCGGGGGCAGGUGUGUG | NM_032741\|AGPAT1 | 5 | 21.392 | 1 | 21 | 163 | 183 |
| GUGGGCGGGGGCAGGUGUGUG | NM_006411\|AGPAT1 | 5 | 19.004 | 1 | 21 | 163 | 183 |
| GUGGGCGGGGGCAGGUGUGUG | NM_001185100\|CD22 | 5 | 7.292 | 1 | 21 | 376 | 396 |
| GUGGGCGGGGGCAGGUGUGUG | NM_020440\|PTGFRN | 5 | 24.034 | 1 | 21 | 2302 | 2323 |
| GUGGGCGGGGGCAGGUGUGUG | NM_001204344\|TNFSF15 | 5 | 11.359 | 1 | 21 | 4453 | 4473 |
| GUGGGCGGGGGCAGGUGUGUG | NM_207386\|SHISA6 | 5 | 12.614 | 1 | 21 | 3790 | 3810 |
| GUGGGCGGGGGCAGGUGUGUG | NM_001185101\|CD22 | 5 | 7.292 | 1 | 21 | 207 | 227 |
| GUGGGCGGGGGCAGGUGUGUG | NM_014620\|HOXC4 | 5 | 0.384 | 1 | 21 | 10 | 30 |
| GUGGGCGGGGGCAGGUGUGUG | NM_001099692\|EIF5AL1 | 5 | 23.625 | 1 | 21 | 721 | 741 |
| GUGGGCGGGGGCAGGUGUGUG | NM_000388\|CASR | 5 | 16.138 | 1 | 21 | 564 | 584 |
| GUGGGCGGGGGCAGGUGUGUG | NM_004760\|STK17A | 5 | 11.229 | 1 | 21 | 727 | 747 |
| GUGGGCGGGGGCAGGUGUGUG | NM_001079871\|HAP1 | 5 | 23.093 | 1 | 21 | 1088 | 1109 |
| GUGGGCGGGGGCAGGUGUGUG | NM_012398\|PIP5K1C | 5 | 22.056 | 1 | 21 | 2920 | 2941 |
| GUGGGCGGGGGCAGGUGUGUG | NM_201274\|MPRIP | 5 | 19.507 | 1 | 21 | 124 | 144 |
| GUGGGCGGGGGCAGGUGUGUG | NM_015134\|MPRIP | 5 | 18.976 | 1 | 21 | 22 | 42 |
| GUGGGCGGGGGCAGGUGUGUG | NM_002193\|INHBB | 5 | 19.144 | 1 | 21 | 1180 | 1202 |
| GUGGGCGGGGGCAGGUGUGUG | NM_173065\|IFNLR1 | 5 | 24.284 | 1 | 21 | 2628 | 2648 |
| GUGGGCGGGGGCAGGUGUGUG | NM_001270614\|DEDD2 | 5 | 13.552 | 1 | 21 | 590 | 610 |
| GUGGGCGGGGGCAGGUGUGUG | NM_001024956\|SC5D | 5 | 18.039 | 1 | 21 | 331 | 351 |
| GUGGGCGGGGGCAGGUGUGUG | NM_170743\|IFNLR1 | 5 | 24.284 | 1 | 21 | 1931 | 1951 |
| GUGGGCGGGGGCAGGUGUGUG | NM_006122\|MAN2A2 | 5 | 21.431 | 1 | 21 | 111 | 131 |
| GUGGGCGGGGGCAGGUGUGUG | NM_014938\|MLXIP | 5 | 16.276 | 1 | 21 | 417 | 437 |
| GUGGGCGGGGGCAGGUGUGUG | NM_022817\|PER2 | 5 | 14.07 | 1 | 21 | 886 | 906 |
| GUGGGCGGGGGCAGGUGUGUG | NM_016577\|RAB6B | 5 | 9.302 | 1 | 21 | 383 | 403 |
| GUGGGCGGGGGCAGGUGUGUG | NM_001003665\|C1orf95 | 5 | 9.313 | 1 | 21 | 5967 | 5988 |
| GUGGGCGGGGGCAGGUGUGUG | NM_004711\|SYNGR1 | 5 | 7.388 | 1 | 21 | 8 | 28 |
| GUGGGCGGGGGCAGGUGUGUG | NM_001113239\|HIPK2 | 5 | 16.561 | 1 | 21 | 9783 | 9803 |
| GUGGGCGGGGGCAGGUGUGUG | NM_021096\|CACNA1I | 5 | 23.359 | 1 | 21 | 2706 | 2726 |
| GUGGGCGGGGGCAGGUGUGUG | NM_033512\|TSPYL5 | 5 | 19.133 | 1 | 21 | 845 | 865 |
| GUGGGCGGGGGCAGGUGUGUG | NM_001040214\|NKAIN2 | 5 | 13.031 | 1 | 21 | 548 | 568 |
| GUGGGCGGGGGCAGGUGUGUG | NM_005937\|MLLT6 | 5 | 21.482 | 1 | 21 | 2253 | 2273 |
| GUGGGCGGGGGCAGGUGUGUG | NM_002501\|NFIX | 5 | 14.893 | 1 | 21 | 3348 | 3368 |
