# Supplementary material for: The sodium new houttuyfonate suppresses NSCLC via activating pyroptosis through TCONS‐14036/miR‐1228‐5p/PRKCDBP pathway
Source: Cell Prolif. 2023 Jan 25;56(7):e13402. doi: 10.1111/cpr.13402 (PMC10334279; doi:10.1111/cpr.13402)
Supplement: Supplementary file 9 — Table S6. The bioinformatic prediction of targets. [file CPR-56-e13402-s009.docx]

**Table S6 Basic information and bioinformatics about TCONS-14036.**

Cufflinks exon 20343070 20343260 . - . gene_id "XLOC_005065"; transcript_id "TCONS_00014036"; exon_number "1"; oId "CUFF.4793.1"; tss_id "TSS9559"; class_code "u";

**Sequence**

GGGGCTCCGCGCGAGGTCAGACTGGGCAGGAGATGCCGTGGACCCCGCCCTTCGGGGAGGGGCCCGGCGGATGCCTCCTTTGCCGGAGCTTGGAACAGACTCACGGCCAGCGAAGTGAGTTCAATGGCTGAGGTGAGGTACCCCGCAGGGGACCTCATAACCCAATTCAGACTACTCTCCTCCGCCCATTT

**miRDB predicted miRNA**

Target Rank Target Score miRNA Name Gene Symbol

1 60 hsa-miR-5192 submission

2 56 hsa-miR-1228-5p submission

3 52 hsa-miR-4680-3p submission

MicroRNA and Target Gene Description:

miRNA Name hsa-miR-5192 miRNA Sequence AGGAGAGUGGAUUCCAGGUGGU

Target Score 60 Seed Location 174 Target Length 191

Custom Target Sequence

1 ggggctccgc gcgaggtcag actgggcagg agatgccgtg gaccccgccc ttcggggagg

61 ggcccggcgg atgcctcctt tgccggagct tggaacagac tcacggccag cgaagtgagt

121 tcaatggctg aggtgaggta ccccgcaggg gacctcataa cccaattcag actactctcc

181 tccgcccatt t

MicroRNA and Target Gene Description:

miRNA Name hsa-miR-1228-5p miRNA Sequence GUGGGCGGGGGCAGGUGUGUG

Previous Name hsa-miR-1228*

Target Score 56 Seed Location 182 Target Length 191

Custom Target Sequence

1 ggggctccgc gcgaggtcag actgggcagg agatgccgtg gaccccgccc ttcggggagg

61 ggcccggcgg atgcctcctt tgccggagct tggaacagac tcacggccag cgaagtgagt

121 tcaatggctg aggtgaggta ccccgcaggg gacctcataa cccaattcag actactctcc

181 tccgcccatt t

MicroRNA and Target Gene Description:

miRNA Name hsa-miR-4680-3p miRNA Sequence UCUGAAUUGUAAGAGUUGUUA

Target Score 52 Seed Location 164 Target Length 191

Custom Target Sequence

1 ggggctccgc gcgaggtcag actgggcagg agatgccgtg gaccccgccc ttcggggagg

61 ggcccggcgg atgcctcctt tgccggagct tggaacagac tcacggccag cgaagtgagt

121 tcaatggctg aggtgaggta ccccgcaggg gacctcataa cccaattcag actactctcc

181 tccgcccatt t

**lnc-CCNB1IP1-1**

Basic information

LNCipedia transcript ID: lnc-CCNB1IP1-1:1

LNCipedia gene ID: lnc-CCNB1IP1-1

Ensembl Gene ID: ENSG00000259001

Ensembl Transcript ID: ENST00000554988

Location (hg38): chr14:20343048-20343685

Strand: -

Class: antisense

Sequence Ontology term: antisense_lncRNA

Transcript size: 638 bp

Exons: 1

Sources: Gencode v13; Ensembl release 68 - Jul 2012; NONCODE v4; Ensembl release 75 - Feb 2014; Ensembl release 83 - Dec 2015; Ensembl release 87 - Dec 2016; Ensembl release 90 - Aug 2017; Ensembl release 92 - Apr 2018

Alternative transcript names: ENST00000554988.1; RPPH1-001; OTTHUMT00000410370.2; NONHSAT035568

Alternative gene names: ENSG00000259001.1; RPPH1; OTTHUMG00000170789.2; ENSG00000259001.2; ENSG00000259001.3; AL355075.4

RNA sequence:

CTCGCCCTGCCGCCGCCGGTGCTCCGTCGCCGCCGCGCCGCCATGGAATTCGAACGCTGACGTCATCAACCCGCTCCAAGGAATCGCGGGCCCAGTGTCACTAGGCGGGAACACCCAGCGCGCGTGCGCCCTGGCAGGAAGATGGCTGTGAGGGACAGGGGAGTGGCGCCCTGCAATATTTGCATGTCGCTATGTGTTCTGGGAAATCACCATAAACGTGAAATGTCTTTGGATTTGGGAATCTTATAAGTTCTGTATGAGACCACTTTTTCCCATAGGGCGGAGGGAAGCTCATCAGTGGGGCCACGAGCTGAGTGCGTCCTGTCACTCCACTCCCATGTCCCTTGGGAAGGTCTGAGACTAGGGCCAGAGGCGGCCCTAACAGGGCTCTCCCTGAGCTTCGGGGAGGTGAGTTCCCAGAGAACGGGGCTCCGCGCGAGGTCAGACTGGGCAGGAGATGCCGTGGACCCCGCCCTTCGGGGAGGGGCCCGGCGGATGCCTCCTTTGCCGGAGCTTGGAACAGACTCACGGCCAGCGAAGTGAGTTCAATGGCTGAGGTGAGGTACCCCGCAGGGGACCTCATAACCCAATTCAGACTACTCTCCTCCGCCCATTTTTGGAAAAAAAAAAAAAAAAAA

**lnc-CCNB1IP1-1:4**

Basic information

LNCipedia transcript ID: lnc-CCNB1IP1-1:4

LNCipedia gene ID: lnc-CCNB1IP1-1

Location (hg38): chr14:20343048-20343409

Strand: -

Class: bidirectional

Sequence Ontology term:

Transcript size: 362 bp

Exons: 1

Sources: FANTOM CAT (stringent)

Alternative transcript names: ENSG00000259001.2|ENST00000554988.1

Alternative gene names:

RNA sequence:

AGGGCGGAGGGAAGCTCATCAGTGGGGCCACGAGCTGAGTGCGTCCTGTCACTCCACTCCCATGTCCCTTGGGAAGGTCTGAGACTAGGGCCAGAGGCGGCCCTAACAGGGCTCTCCCTGAGCTTCGGGGAGGTGAGTTCCCAGAGAACGGGGCTCCGCGCGAGGTCAGACTGGGCAGGAGATGCCGTGGACCCCGCCCTTCGGGGAGGGGCCCGGCGGATGCCTCCTTTGCCGGAGCTTGGAACAGACTCACGGCCAGCGAAGTGAGTTCAATGGCTGAGGTGAGGTACCCCGCAGGGGACCTCATAACCCAATTCAGACTACTCTCCTCCGCCCATTTTTGGAAAAAAAAAAAAAAAAAA
